# Evolutionary dynamics of residual disease in human glioblastoma

**DOI:** 10.1093/annonc/mdy506

**Published:** 2018-11-19

**Authors:** I Spiteri, G Caravagna, G D Cresswell, A Vatsiou, D Nichol, A Acar, L Ermini, K Chkhaidze, B Werner, R Mair, E Brognaro, R G W Verhaak, G Sanguinetti, S G M Piccirillo, C Watts, A Sottoriva

**Affiliations:** 1 Evolutionary Genomics & Modelling Lab, Centre for Evolution and Cancer, The Institute of Cancer Research, London; 2 Centre for Evolution and Cancer, The Institute of Cancer Research, London; 3 Department of Clinical Neurosciences, University of Cambridge, Cambridge, UK; 4 Department of Neurosurgery, S. Maria Della Misericordia Hospital, Rovigo, Italy; 5 Jackson Laboratory for Genomic Medicine, Farmington, USA; 6 School of Informatics, University of Edinburgh, Edinburgh, UK; 7 Division of Hematology and Oncology, Department of Internal Medicine, University of Texas Southwestern Medical Center, Dallas, USA; 8 Institute of Cancer Genome Sciences, University of Birmingham, Birmingham, UK

**Keywords:** glioblastoma, tumour margin, sub-ventricular zone, cancer evolution, phylogenetics

## Abstract

**Background:**

Glioblastoma is the most common and aggressive adult brain malignancy against which conventional surgery and chemoradiation provide limited benefit. Even when a good treatment response is obtained, recurrence inevitably occurs either locally (∼80%) or distally (∼20%), driven by cancer clones that are often genomically distinct from those in the primary tumour. Glioblastoma cells display a characteristic infiltrative phenotype, invading the surrounding tissue and often spreading across the whole brain. Cancer cells responsible for relapse can reside in two compartments of residual disease that are left behind after treatment: the infiltrated normal brain parenchyma and the sub-ventricular zone. However, these two sources of residual disease in glioblastoma are understudied because of the difficulty in sampling these regions during surgery.

**Patient and methods:**

Here, we present the results of whole-exome sequencing of 69 multi-region samples collected using fluorescence-guided resection from 11 patients, including the infiltrating tumour margin and the sub-ventricular zone for each patient, as well as matched blood. We used a phylogenomic approach to dissect the spatio-temporal evolution of each tumour and unveil the relation between residual disease and the main tumour mass. We also analysed two patients with paired primary-recurrence samples with matched residual disease.

**Results:**

Our results suggest that infiltrative subclones can arise early during tumour growth in a subset of patients. After treatment, the infiltrative subclones may seed the growth of a recurrent tumour, thus representing the ‘missing link’ between the primary tumour and recurrent disease.

**Conclusions:**

These results are consistent with recognised clinical phenotypic behaviour and suggest that more specific therapeutic targeting of cells in the infiltrated brain parenchyma may improve patient’s outcome.

Key Message

In this study, we investigate the evolution of residual disease in glioblastoma that is left behind after surgery, in particular cancer cells in the infiltrative tumour margin and in the sub-ventricular zone. We found that residual disease samples contained early precursor cancer clones that were involved in the development of the disease and may contribute to recurrence.

## Introduction

Glioblastoma (GB) is a lethal brain cancer against which effective therapeutic options are lacking [1]. The disease is characterised by variegated clinical phenotypes [2–4] and intra-tumour heterogeneity (ITH) [5–7]. The disease aetiology and clinical course have distinct features compared with other cancers. Unlike other solid tumours, GB rarely metastasises outside the brain, but it invariably recurs, limiting the median survival to approximately 14 months [1]. In approximately 80% of aggressively treated patients, disease progression/recurrence occurs within 2 cm of the resection margin. In the remaining patients, even when complete surgical removal of the primary lesion was possible, the tumour recurs distally [6–8] and even drastic hemispherectomy procedures fail to eradicate the disease [9]. Cancer cells from these distal recurrent lesions, despite sharing a common ancestor with the primary tumour, are often genomically distinct [6–8]. Moreover, at diagnosis, GB already displays a characteristic infiltrative phenotype, invading the surrounding brain tissue and often diffusely infiltrating the whole brain [10]. We have shown previously that malignant clones present in the sub-ventricular zone (SVZ), a known neural stem cell germinal niche, often contain tumour precursor cells [11], a finding that has been recently corroborated [12]. Indeed, infiltration is ubiquitous in GB, with cells migrating through diverse regions of the brain microenvironment including white matter tracts [13] and blood vessels [14]. In addition, up to 10% of GB cases present as multifocal disease at diagnosis [15], a rare occurrence in other solid tumours.

**Figure 1.**
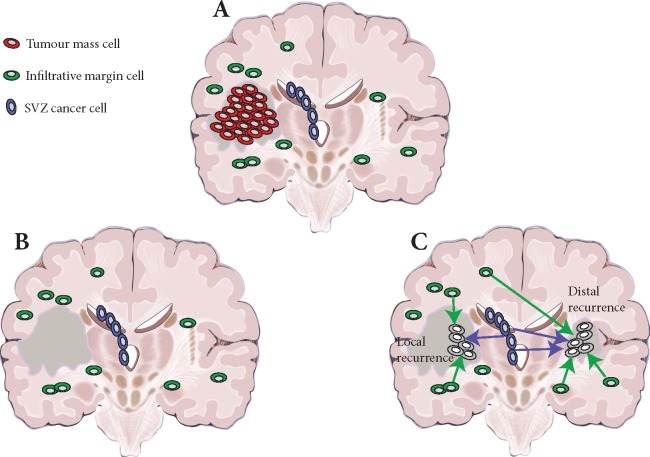
Residual disease in glioblastoma. (A) At surgery, only the primary tumour mass (red) is removed (in dark grey the resection cavity). (B) However, infiltrative cells in the normal brain parenchyma (green) and sub-ventricular zone (SVZ) (blue) are left behind. (C) Residual glioblastoma cells infiltrated throughout the brain can give rise to relapse, both locally and distally.

The accumulating clinical and genomic evidence suggests that infiltration may be a very early event in GB development, so that no matter how early a cancer is detected, it has already spread to distal regions of the brain, including the normal brain parenchyma and the SVZ (Figure 1A). After treatment (Figure 1B), infiltrative cells in the brain parenchyma and in the SVZ can drive relapse. Thus, even in the case of optimal surgery and when the tumour mass has been macroscopically resected, residual disease will trigger new growth, giving rise to recurrence, either locally or distally (Figure 1C). We argue that the recent seminal studies carried out on primary-recurrent-matched GB samples [6–8] point at the infiltrative population as the ‘missing link’, connecting the primary and the recurrent malignant clone in the evolution of the disease.

## Methods

### Patient cohort and samples

**Figure 2.**
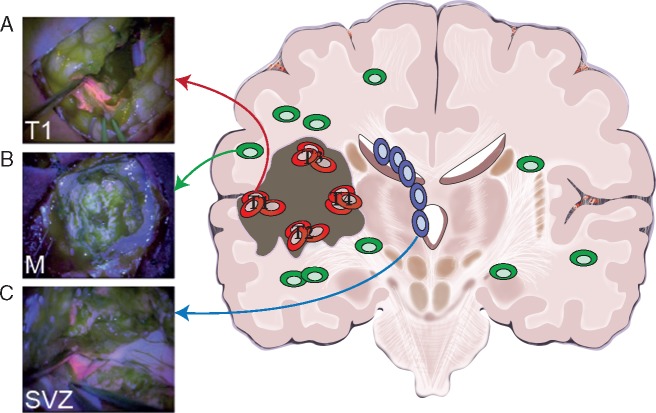
Study design: multi-region tumour and residual disease sampling. (A) The large majority of patients present at diagnosis with a large tumour mass that is positive for 5-ALA fluorescence. In this study, we collected multiple spatially separated regions of the tumour mass (four to six regions per tumour in nine patients), as well as matched primary-relapse samples in two patients. (B) Extensive infiltration is also present in the surrounding normal brain but cancer cells are so sparse beyond the resection margin that do not appear fluorescent. Samples from the non-fluorescent infiltrative margin were collected from 9/11 patients. From paired primary-recurrent patients, we collected matched margin from the primary tumour and another margin sample from the relapsed neoplasm. (C) In a subset of patients, disease is also found in the sub-ventricular zone (SVZ), which appears fluorescent and contains malignant clones. We collected one to three samples of the SVZ from all patients, including matched SVZ in primary and relapsed tumours. Through surgery and chemo-radiation, it is possible to extensively remove the primary tumour but treatment is unlikely to completely remove the infiltrative disease, nor cancer cells in the SVZ. Those represent the majority of residual disease in glioblastoma.

Sixty-nine tissue samples were collected from neurosurgical fluorescence-guided resections carried out on 10 IDH1 wildtype GB patients and one IDH1 mutant anaplastic astrocytoma patient (see supplementary Table S1, available at *Annals of Oncology* online for clinical information). Between five and nine multiple samples from the tumour mass (T, at least 1 cm apart), SVZ and infiltrative margin (M) areas were collected from each patient (Figure 2). Tumour mass samples were numbered as the surgery progressed and hence T4 samples tend to be deeper into the resection cavity than T1 samples. SVZ samples are taken after T4. In the case of the two primary/recurrence cases, three samples (T, SVZ and M) were taken during the primary and secondary surgical resections for a total of six specimens per patient. Thirty 10-μm cryosections were taken from each frozen tissue for DNA extraction using the DNeasy Blood & Tissue kit (Qiagen, Hilden, Germany). Patient informed consent was obtained and tissue collection/storage protocols were compliant with the UK Human Tissue Act 2004 and approved by the Local Regional Ethics Committee (LREC ref 04/Q0108/60). No difference in 5-ALA labelling capacity was observed between patients.

### Whole-exome and targeted sequencing

**Figure 3.**
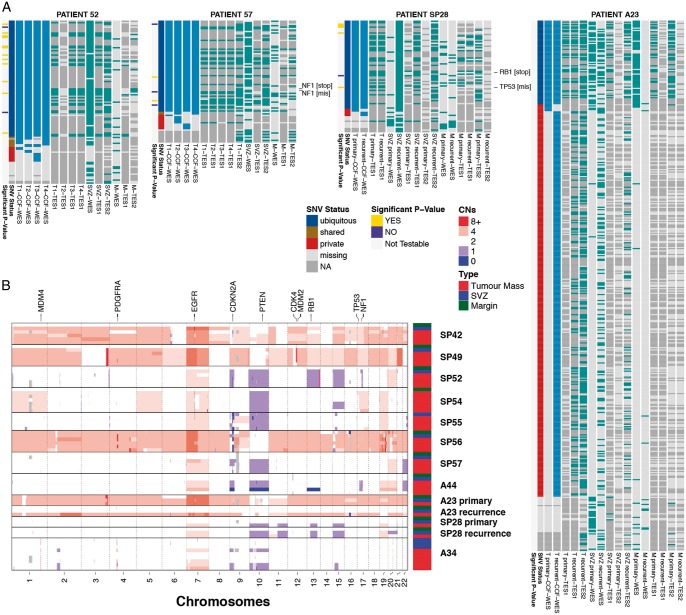
Multi-region genomic profiles of glioblastoma residual disease. (A) For four representative patients we report the cancer cell fractions (>80%) for the tumour mass samples and presence/absence of mutation in all the residual disease samples (see supplementary Figure S1 for all cases, supplementary Table S2 for purity and supplementary data, available at *Annals of Oncology* online for sub-ventricular zone (SVZ) calls). Putative SNV driver events are annotated. WES, whole-exome sequencing; TES1, targeted amplicon sequencing panel 1; TES2, targeted exome capture sequencing panel 2; T1…4, tumour mass sample; SVZ, sub-ventricular zone; M, margin. (B) Digital copy number alterations are reported for each sample (see supplementary Figure S2 and Table S3, available at *Annals of Oncology* online for details).

Between 100 and 300 ng of DNA from each of the 69 tumour specimens and 11 blood samples were used for whole-exome sequencing using the Agilent SureSelectXT Human All Exon V5 Kit (Agilent Technologies, Santa Clara, CA, US). Median coverage was ×157 (min. ×108, max. ×187). A custom targeted sequencing panel for 891 single nucleotide variants (SNVs) (covered by a total of 5090 amplicons) identified from the exome sequencing data was designed using Agilent’s Haloplex technology (TES1). In addition, we designed a separate Agilent SureSelect XT2 capture panel to specifically validate 1054 SNVs found in the M and SVZ samples across all patients (TES2). Both amplicon (TES1) and targeted capture (TES2) libraries were sequenced on an Illumina HiSeq2500 obtaining a median coverage of ×4050 and ×1128, respectively, in reported variants (Figure 3A and supplementary Figure S1, available at *Annals of Oncology* online). Copy number alterations per sample are reported in Figure 3B. See supplementary Material and methods, available at *Annals of Oncology* online for details about bioinformatics analysis.

## Results

### ITH in the tumour mass and residual disease

We carried out fluorescence-guided multi-region sampling of different regions from primary tumours (T1, T2, T3, …; Figure 2A) and also collected samples from the infiltrative margin M (*n *=* *11; Figure 2B) and the SVZ (*n *=* *15; Figure 2C). The margin is defined by non-fluorescent tissue beyond the fluorescent tumour mass. We reported previously that this area appears histologically as normal brain and is composed by only 5%–10% of tumour cells [16]. The tumour mass and SVZ samples displayed high tumour content (median 58.5% and 22.1%, respectively). The SVZ samples are fluorescent and we demonstrated contain cancer clones [11]. Clinical and follow-up information, as well as imaging was available (supplementary Table S1, available at *Annals of Oncology* online). Samples from the tumour mass and SVZ from 7/11 patients were common to our previous studies, for which we had carried out microarray copy number profiling and gene expression alone [5, 11]. The margin samples are presented here for the first time.

In the present study, multi-region whole-exome sequencing (see supplementary Material and methods, available at *Annals of Oncology* online) identified extensive ITH at the level of SNVs. Heterogeneity at the level of SNV putative drivers (from ref. [4]) was evident in 5/11 patients, especially in EGFR, PIK3R1 and TP53 (Figure 3A and supplementary Figure S1, available at *Annals of Oncology* online). Copy number profiles inferred from the whole-exome sequencing confirmed the heterogeneity levels reported in previous studies (Figure 3B and supplementary Figure S2, available at *Annals of Oncology* online). Copy number events were highly recurrent, especially EGFR amplification (SP42, SP54, SP55, A23 and A34), chromosome 10 loss containing PTEN (all patients), and CDKN2A homozygous deletion (SP42, SP52, SP55, SP56, SP57 and A44), corroborating the findings from large scale studies [2]. Our custom-targeted panel TES1 (see supplementary Material and methods, available at *Annals of Oncology* online) confirmed the results from exome sequencing (Figures 3A and supplementary Figure S1, available at *Annals of Oncology* online). See supplementary Table S2, available at *Annals of Oncology* online for purity estimations, supplementary Table S3, available at *Annals of Oncology* online for copy number states and supplementary Data, available at *Annals of Oncology* online for VCF files with SNV calls.

Because the infiltrative margin (M) samples consist of scattered cancer cells in the surrounding normal brain, the purity of those samples was expectedly low (5%–10%). Purity is a confounding factor for calling mutations, leading to possible false-negatives that may impact the phylogenetic analysis [17]. To tackle this problem, we designed a second targeted sequencing panel (TES2) specifically to validate whether mutations that were present in all the other tumour samples were really absent from the infiltrative areas (indicating the margin or SVZ as an ancestral subclone). This panel confirmed that several mutations that appeared truncal to the tumour mass (putative truncal) were not present in the margin sample (Figure 3A and supplementary Figure S1, available at *Annals of Oncology* online, *TES2* panel). In addition, to further support our results, we developed a statistical method to test whether genomic variants in targeted sequencing that are not found in the margin are likely to be truly absent, rather than being false negatives (i.e. in the second case, if there is no power to determine with reasonable certainty that the mutation is not present).

**Figure 4.**
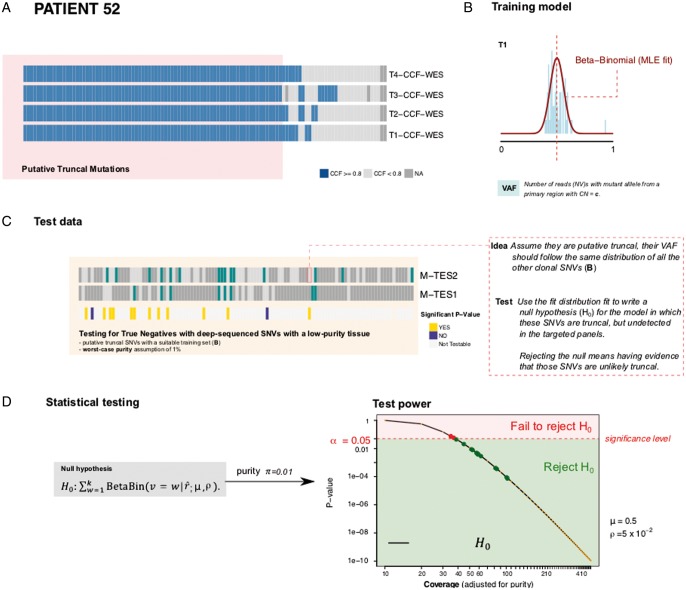
Testing the absence of putative truncal mutations in the infiltrative margin (representative case SP52). (A) We detected single nucleotide variants (SNVs) using joint-sample variant calling from whole-exome sequencing (WES). We selected SNVs that had cancer cell fraction (CCF) ≥ 0.8 in all tumour mass samples (T1, T2, …) and the same CNA status across all T samples. We call these ‘putative truncal’ SNVs. If cancer cells in M developed from T, then these mutations are ‘truly truncal’ and should be detected also in M. However, calling these mutations in the margin might be confounded by the low purity of margin samples. (B) From read counts of selected SNVs, we train for every sample a beta-binomial model of expected variant allele frequency (VAF), accounting for tumour purity and copy number status. This model describes, for each such SNV, the expected number of reads with the variant allele as a function of sample purity (i.e. we can predict how many mutant reads we expect to find in a sample like M, at purity 5%). (C) We use deep-sequencing data from targeted panels TES1 and TES2 to identify which putative truncal SNVs were not detected in the margin sample by any assay (missing SNVs). Based on the beta-binomial trained model, we created a statistical test for the null hypothesis that these mutations are truly truncal in the tumour (and hence present also in M) but remain undetected in M due to low purity. (D) Based on the expectation and the depth of coverage achieved for each tested mutation, we can calculate a *P*-value under the null. Rejecting the null means that we have evidence for the fact that these SNVs are not truly truncal, and that they are missing in the margin. This provides further evidence that the margin is ancestral to the tumour mass. The power of the test increases with higher coverage; we used a conservative setting of worst-case purity with πvalue (1% tumour, 99% normal) for the test, and corrected it for multiple testing via Bonferroni.

Our test scans mutations that were identified as ‘putatively truncal’ from the tumour mass samples (T1, T2, …). These mutations have high cancer cell fraction (CCF) in all T samples (Figure 4A, representative example of patient SP52). We reasoned that if the cancer subclones in the margin were just infiltrative cells deriving from the tumour mass—rather than an early ancestor—then ‘putative truncal’ mutations should also be found in the margin (‘truly truncal’). However, a failure to identify these mutations in the margin, despite the very high depth of sequencing of targeted panels, may occur as a false-negative owing to low purity. Similar to previous approaches [18], our statistical method accounts for the confounding factor of purity, and tests the null hypothesis that ‘putative truncal’ mutations are present in the margin, but miss to be detected in the margin. Rejecting the null indicates that these mutations are unlikely to be present in M.

We first fit a beta-binomial distribution to the set of putative truncal mutations, separated by copy number state (Figure 4B, see supplementary Material and methods, available at *Annals of Oncology* online for details). This provides the expected variant allele frequency of a mutation in a high purity sample, such as T1–4. This model allows calculation of the expected frequency of a mutant given any purity value, for example the 5% purity of the margin sample. We then examine the whole-exome and targeted sequencing data (TES1 and TES2) from margin samples and consider those putative truncal SNVs that were not detected by any assay (Figure 4C). Given the coverage achieved at the locus of the missing mutation, and the beta-binomial model trained on putative truncal SNVs, we can calculate the likelihood of the data assuming the null is true (i.e. the mutation is *truly truncal* but 0 mutant reads are found at the locus, for a given purity). Application of our method to our dataset revealed that a considerable proportion of testable mutations (see supplementary Material and methods, available at *Annals of Oncology* online for details) were unlikely to be present in the margin (*P*<0.05 using Bonferroni correction), even when we assumed the margin purity to be as low as 1% (Figure 4D). These results indicate that these SNVs are likely to be absent in M, and hence cannot be truncal. The method is potentially applicable to any genomic dataset to test true negatives (see supplementary Material and methods and Figure S3, available at *Annals of Oncology* online). The results of the test for all patients are reported in Figure 3A and supplementary Figure S1, available at *Annals of Oncology* online, left hand side of each heatmap. This allowed us to carry out a more reliable phylogenetic analysis for each patient that included residual disease in the SVZ and the infiltrative margin M.

### Evolutionary trajectories suggest early ancestor clones within residual disease

**Figure 5.**
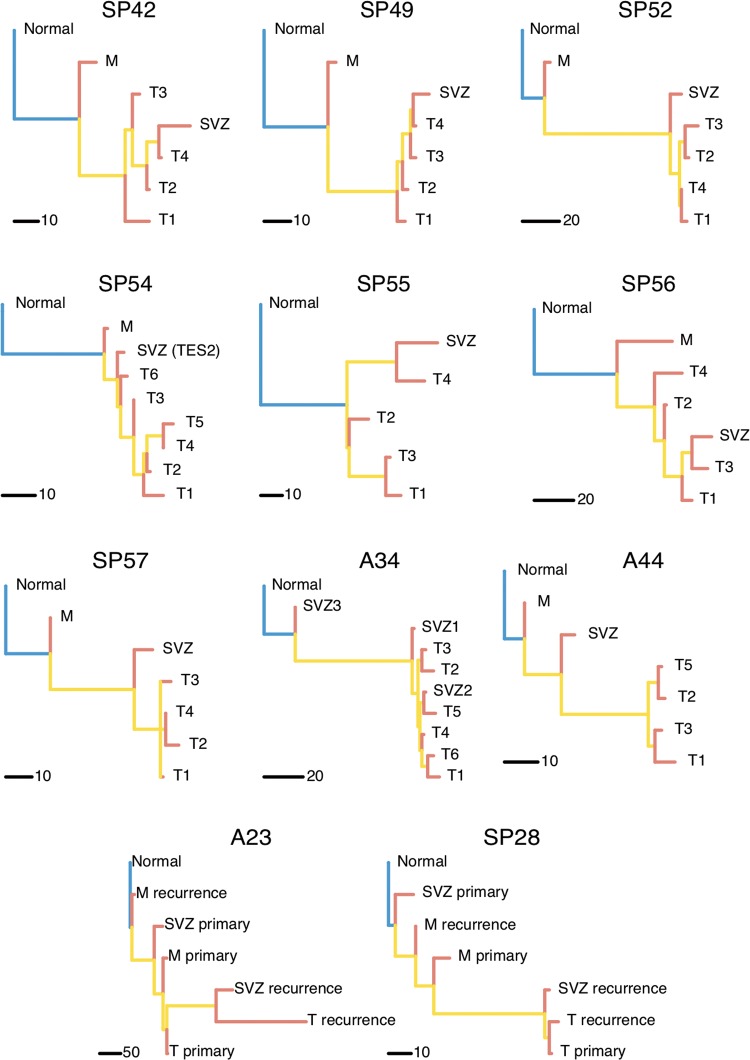
Phylogenetic reconstruction indicates residual disease subclones may arise early. Phylogenetic trees built with whole-exome sequencing (WES) data and excluding mutations that do not pass our test show the infiltrative margin sample at the top of the phylogeny, suggesting it contains cancer clones that occur early during tumour growth. In 6/11 samples the sub-ventricular zone (SVZ) appears as an early subclone as well. Often the phylogeny recapitulates the spatial structure of the tumour, where T1, T2, … T4 samples are taken in this order as the tumour resection extends deeper into the brain. Matched samples from M and SVZ in paired primary-relapse cases A23 and SP28 show the role of residual disease in the development of glioblastoma recurrence.

Residual disease samples diverged early from the rest of the tumour mass in the majority of patients for which M samples were available, with particular evidence for SP49, SP52, SP57, A44, SP28 for which we could apply our test (Figure 5, testable mutations that did not pass our test were excluded from the phylogeny). Importantly, residual disease was also found at recurrence, demonstrating the presence of a reservoir of cancer cells in the infiltrative margin at relapse (patients A23 and SP28). In case A23, the primary tumour mass appeared to have originated from earlier cancer cell lineages located in the margin M and SVZ collected at primary resection. However, at the time of relapse, whereas both T and SVZ appear to have acquired additional new mutations (long branch), the ‘M recurrence’ lineage has remained similar to the primary tumour. A comparable pattern is observed in SP28 where ‘M recurrence’ also shows as an early residual clone present at relapse. Hence, early ancestral clones are present both at primary and recurrence and are not generally resected. We do acknowledge that the bulk tumour mass at relapse can also be driven by incomplete resection of the primary tumour due to the neoplastic tissue extending to vital parts of the brain that cannot be removed. We note that A23 and SP28 were local recurrences and more residual disease samples from distal recurrent tumours will need to be collected in the future.

As a whole, this analysis indicates that subclones present in M may arise early during tumour growth. Moreover, in 6/11 patients, the SVZ appeared also as an early ancestor, as we previously reported [11] and as recently confirmed [12]. See supplementary Figure S4, available at *Annals of Oncology* online for bootstrapping values. We do acknowledge however that in those branches some mutations may be missing due to limits of detectability. To validate further these results, we also carried out single-allele methylation molecular clock analysis [19] on the same samples for a subset of patients, in particularly those where we had primary-recurrence pairs (supplementary Figure S5, available at *Annals of Oncology* online). Methylation molecular clock haplotyping is a single-molecule approach that allows reading the status of single CpGs in CpG island from the same DNA molecule. We have shown that some of these CpG island loci can be used for phylogenetic reconstruction, both colorectal cancer and GB [11, 19]. Importantly, because this assay is ‘single-molecule’, alleles that come from non-proliferative normal cells (e.g. normal contamination from neurons in the margin samples) can be discarded because of their low methylation status. Therefore, this analysis does not suffer from the problems of tumour purity of the exome analysis (see supplementary Material and methods, available at *Annals of Oncology* online). Eliminating the methylation haplotypes coming from non-cancer cells allows reconstructing the tumour phylogeny orthogonally with respect to the exome trees. The results strikingly confirmed the structure of the phylogenetic trees inferred from exome sequencing, thus corroborating the results in Figure 5.

Interestingly, histopathology reports (pre WHO 2016 revision) are congruent with these phylogenetic data in multiple cases, for example SP49 GB with low-grade areas, SP52 and SP28 GB with oligodendroglial component. Together these data indicate a less aggressively proliferative phenotype at early stages of the evolution of the malignancy. After the primary tumour has been treated with radio- and chemotherapy, the quiescent residual subclones may trigger new growth and further clonal evolution, producing the divergence that has been observed by us and others between primary and recurrent samples in GB. This interpretation of the data is consistent with an early onset of tumour cell infiltration. Residual ancestral disease present in the SVZ and in the infiltrative margin is the source of the inevitable relapse that occurs in GB patients. This model is also consistent with the high incidence of multifocal lesions and the accumulating evidence of evolutionary divergence that is emerging from genomic data [6, 7].

## Discussion

A key to understanding cancer is not just exposing ITH, the natural process that underlines clonal evolution, but also to understand such heterogeneity in a way that is clinically relevant and therapeutically tractable. An important aspect of genomic ITH is that it embeds the evolutionary history of the tumour, a fundamental biological element that cannot be directly measured in humans. Nevertheless, inferring and understanding that history may be critical in developing a rationale for combinatorial therapeutics [20].

Specifically, in this study, we leveraged the spatio-temporal decomposition of the clonal architecture of the tumour to understand the link between subclones in the main tumour mass and in residual disease left behind in the surrounding brain parenchyma and SVZ following surgery. This residual disease is a key factor contributing to GB treatment failure because of resistance to radiation and alkylating chemotherapy coupled with an inherent ability to seed re-growth. Therefore, the main message of this study is that residual disease and not just the main tumour mass [5–8, 21, 22] must be investigated in depth from the point of view of tumour evolution if we are to understand how treatment-resistant disease develops. We acknowledge that to study the mechanisms that link residual disease to tumour relapse, additional analysis is needed, especially of primary-recurrence pairs from distal relapses where also SVZ and M would be collected. However, this remains a technical and ethical challenge.

This observation presents a challenge to the GB research community to develop the tools and strategies needed to collect and robustly analyse difficult samples from residual disease areas in prospective cohorts. Due to the limited number of patients in this study, additional analyses on larger cohorts are necessary to validate these findings. We acknowledge that analysing sparse cancer cells in the margin remains challenging, even with the most advanced sequencing and bioinformatics approaches currently available. Further work is also needed to improve purification of margin samples, which is not currently possible due to lack of reliable markers to sort GB cells. Therefore, new efforts of collecting infiltrative cells that lay distant from the main tumour mass will be needed to study residual disease with more accuracy. To do this, post-mortem efforts such as the PEACE study (Postohumous Evaluation of Advanced Cancer Environment) are likely to play a key role in revealing the biology of infiltrative disease in GB. 

## Supplementary Material

Supplementary Data
