# Supplementary material for: Evolutionary dynamics of residual disease in human glioblastoma
Source: Ann Oncol. 2018 Nov 19;30(3):456–63. doi: 10.1093/annonc/mdy506 (PMC6442656; doi:10.1093/annonc/mdy506)
Supplement: Supplementary Data [file mdy506_supp.zip › mdy506-suppl_data/mdy506_Supplementary_Fig_S3.pdf]

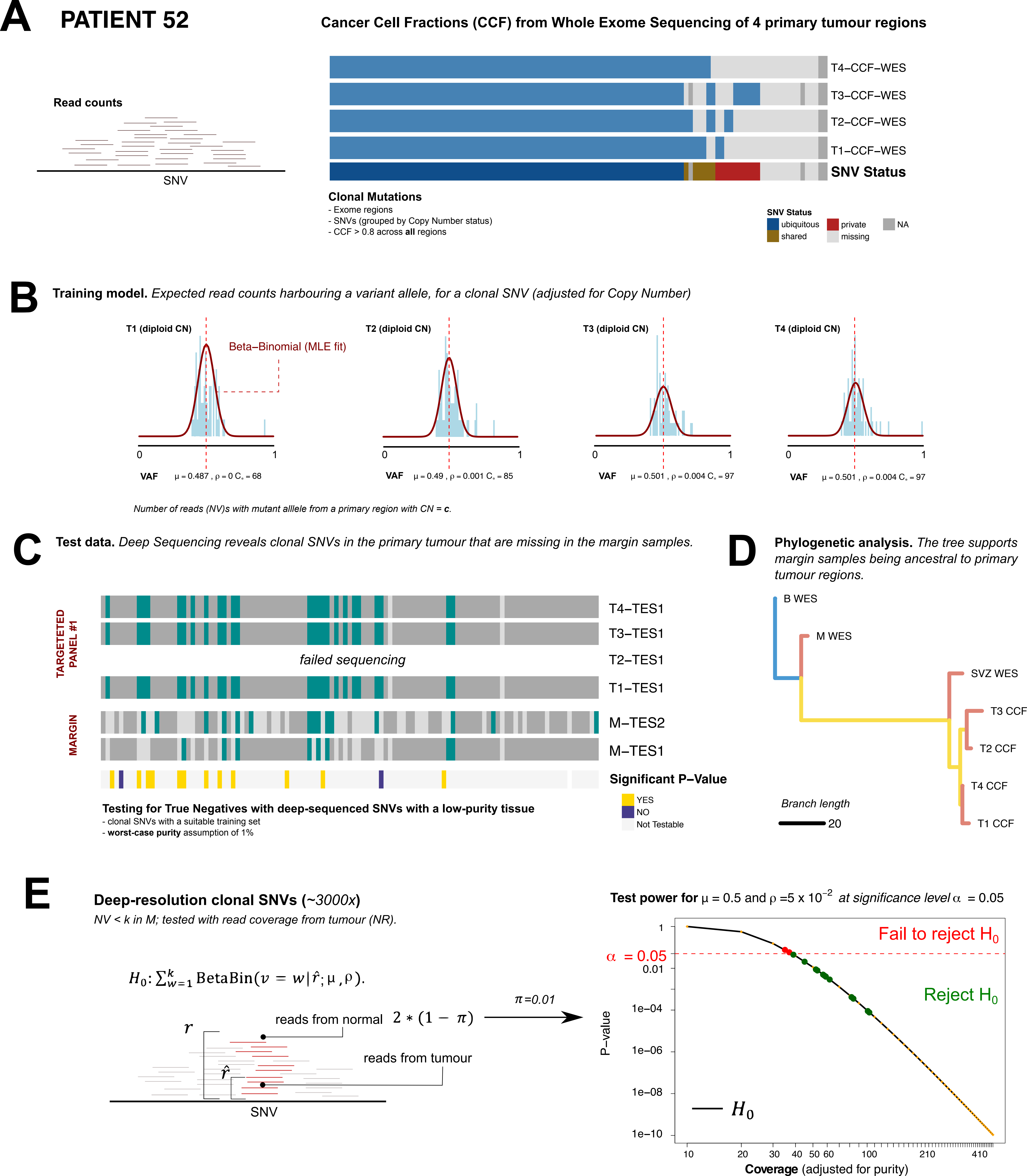

A
PATIENT 52
Cancer Cell Fractions (CCF) from Whole Exome Sequencing of 4 primary tumour regions
Read counts
SNV
T4–CCF–WES
T3–CCF–WES
T2–CCF–WES
T1–CCF–WES
SNV Status
Clonal Mutations
- Exome regions
- SNVs (grouped by Copy Number status)
- CCF > 0.8 across all regions
SNV Status
ubiquitous
shared
private
missing
NA
B
Training model. Expected read counts harbouring a variant allele, for a clonal SNV (adjusted for Copy Number)
T1 (diploid CN)
Beta–Binomial (MLE fit)
T2 (diploid CN)
T3 (diploid CN)
T4 (diploid CN)
0
1
VAF
μ = 0.487 , ρ = 0 C* = 68
μ = 0.49 , ρ = 0.001 C* = 85
μ = 0.501 , ρ = 0.004 C* = 97
μ = 0.501 , ρ = 0.004 C* = 97
Number of reads (NV)s with mutant alllele from a primary region with CN = c.
C
Test data. Deep Sequencing reveals clonal SNVs in the primary tumour that are missing in the margin samples.
TARGETETED PANEL #1
T4–TES1
T3–TES1
failed sequencing
T2–TES1
T1–TES1
MARGIN
M–TES2
M–TES1
Significant P–Value
YES
NO
Not Testable
Testing for True Negatives with deep-sequenced SNVs with a low-purity tissue
- clonal SNVs with a suitable training set
- worst-case purity assumption of 1%
D
Phylogenetic analysis. The tree supports margin samples being ancestral to primary tumour regions.
B WES
M WES
SVZ WES
T3 CCF
T2 CCF
T4 CCF
T1 CCF
Branch length
20
E
Deep-resolution clonal SNVs (~3000x)
NV < k in M; tested with read coverage from tumour (NR).
$H_0: \sum_{w=1}^{k} \mathrm{BetaBin}(v = w \mid \hat{r}; \mu, \rho)$.
r
reads from normal
$2 * (1 - \pi)$
π=0.01
$\hat{r}$
reads from tumour
SNV
Test power for μ = 0.5 and ρ = 5 x 10^-2 at significance level α = 0.05
Fail to reject H0
α = 0.05
Reject H0
1
0.01
1e–04
1e–06
1e–08
1e–10
P–value
H0
10
20
30
40
50
60
100
210
410
Coverage (adjusted for purity)
