# Supplementary material for: Evolutionary dynamics of residual disease in human glioblastoma
Source: Ann Oncol. 2018 Nov 19;30(3):456–63. doi: 10.1093/annonc/mdy506 (PMC6442656; doi:10.1093/annonc/mdy506)
Supplement: Supplementary Data [file mdy506_supp.zip › mdy506-suppl_data/mdy506_Supplementary_Fig_S2.pdf]

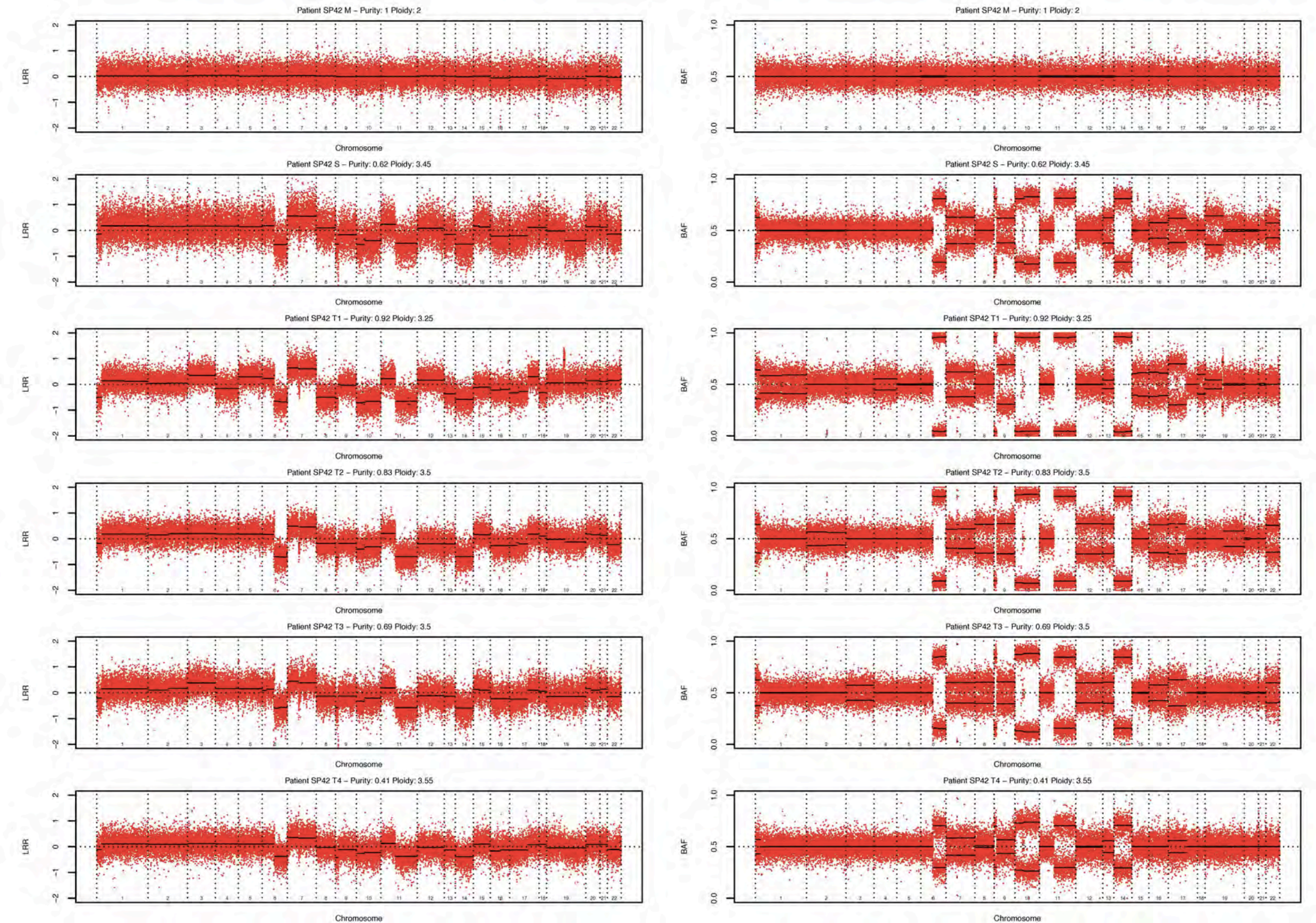
Patient SP42 M – Purity: 1 Ploidy: 2
Patient SP42 S – Purity: 0.62 Ploidy: 3.45
Patient SP42 T1 – Purity: 0.92 Ploidy: 3.25
Patient SP42 T2 – Purity: 0.83 Ploidy: 3.5
Patient SP42 T3 – Purity: 0.69 Ploidy: 3.5
Patient SP42 T4 – Purity: 0.41 Ploidy: 3.55
LRR
BAF
Chromosome

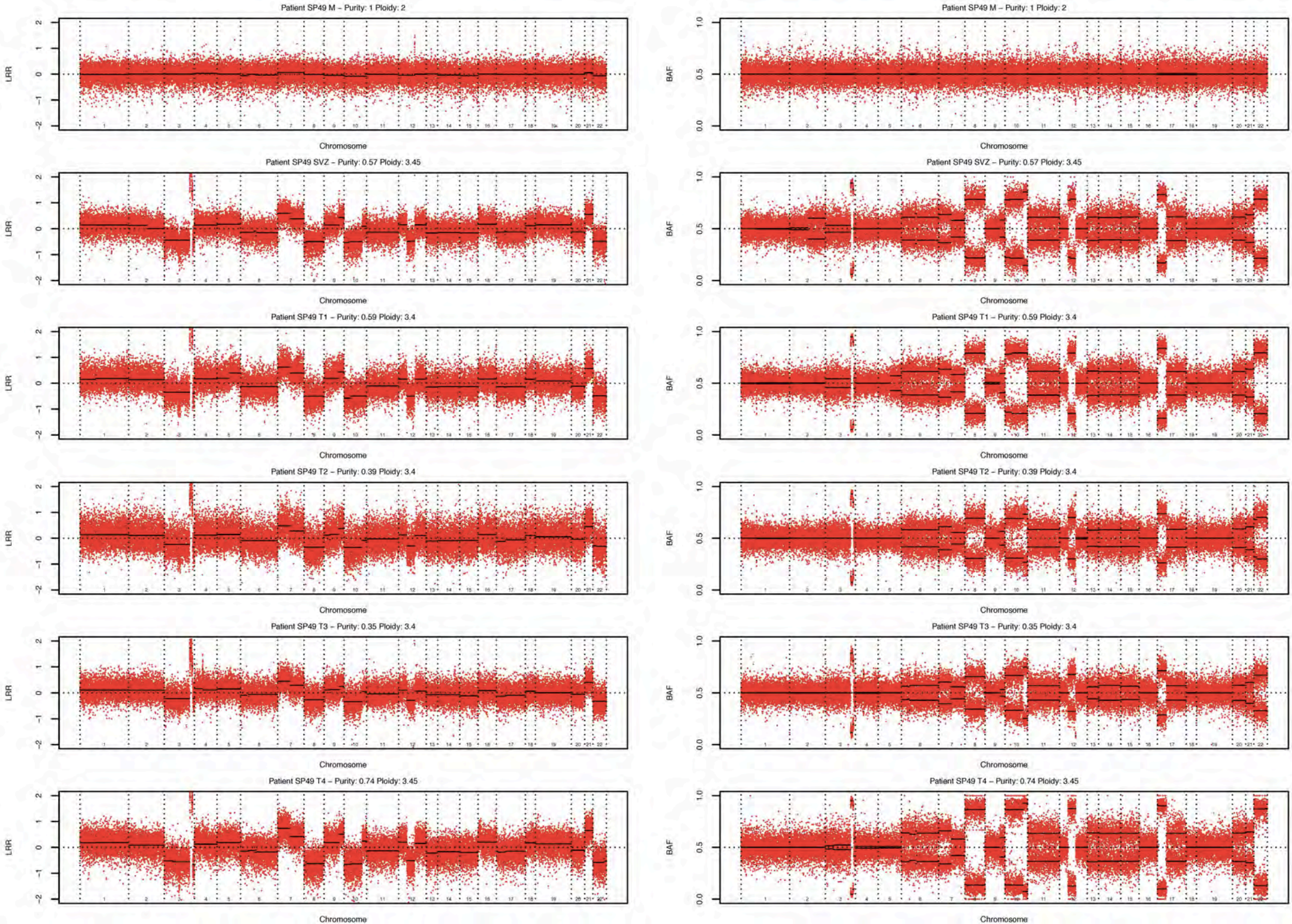
Patient SP49 M – Purity: 1 Ploidy: 2
Patient SP49 SVZ – Purity: 0.57 Ploidy: 3.45
Patient SP49 T1 – Purity: 0.59 Ploidy: 3.4
Patient SP49 T2 – Purity: 0.39 Ploidy: 3.4
Patient SP49 T3 – Purity: 0.35 Ploidy: 3.4
Patient SP49 T4 – Purity: 0.74 Ploidy: 3.45
LRR
BAF
Chromosome

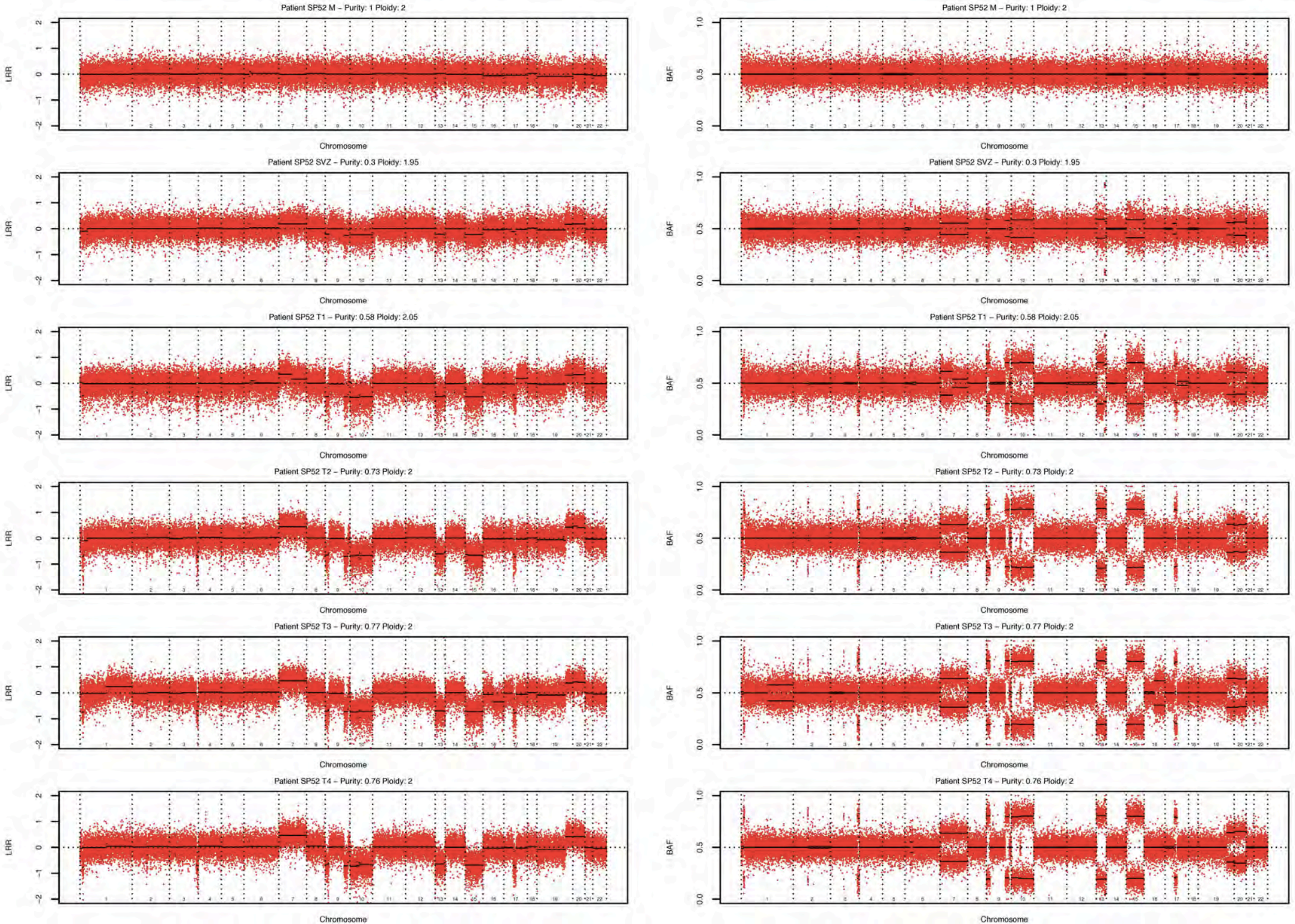
Patient SP52 M – Purity: 1 Ploidy: 2
LRR
Chromosome
Patient SP52 M – Purity: 1 Ploidy: 2
BAF
Chromosome
Patient SP52 SVZ – Purity: 0.3 Ploidy: 1.95
LRR
Chromosome
Patient SP52 SVZ – Purity: 0.3 Ploidy: 1.95
BAF
Chromosome
Patient SP52 T1 – Purity: 0.58 Ploidy: 2.05
LRR
Chromosome
Patient SP52 T1 – Purity: 0.58 Ploidy: 2.05
BAF
Chromosome
Patient SP52 T2 – Purity: 0.73 Ploidy: 2
LRR
Chromosome
Patient SP52 T2 – Purity: 0.73 Ploidy: 2
BAF
Chromosome
Patient SP52 T3 – Purity: 0.77 Ploidy: 2
LRR
Chromosome
Patient SP52 T3 – Purity: 0.77 Ploidy: 2
BAF
Chromosome
Patient SP52 T4 – Purity: 0.76 Ploidy: 2
LRR
Chromosome
Patient SP52 T4 – Purity: 0.76 Ploidy: 2
BAF
Chromosome

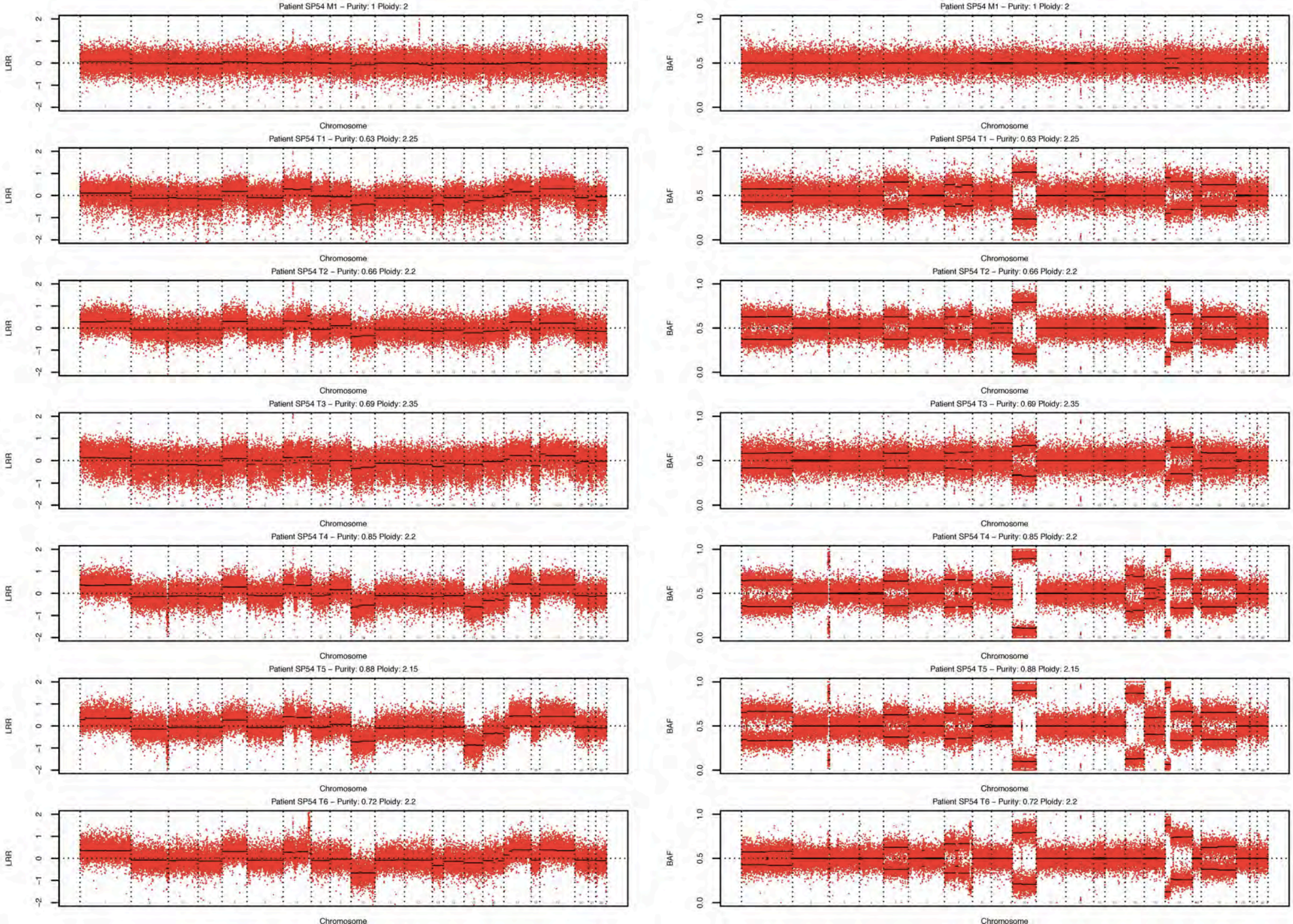
Patient SP54 M1 – Purity: 1 Ploidy: 2
LRR
BAF
Chromosome
Patient SP54 T1 – Purity: 0.63 Ploidy: 2.25
Patient SP54 T2 – Purity: 0.66 Ploidy: 2.2
Patient SP54 T3 – Purity: 0.69 Ploidy: 2.35
Patient SP54 T4 – Purity: 0.85 Ploidy: 2.2
Patient SP54 T5 – Purity: 0.88 Ploidy: 2.15
Patient SP54 T6 – Purity: 0.72 Ploidy: 2.2

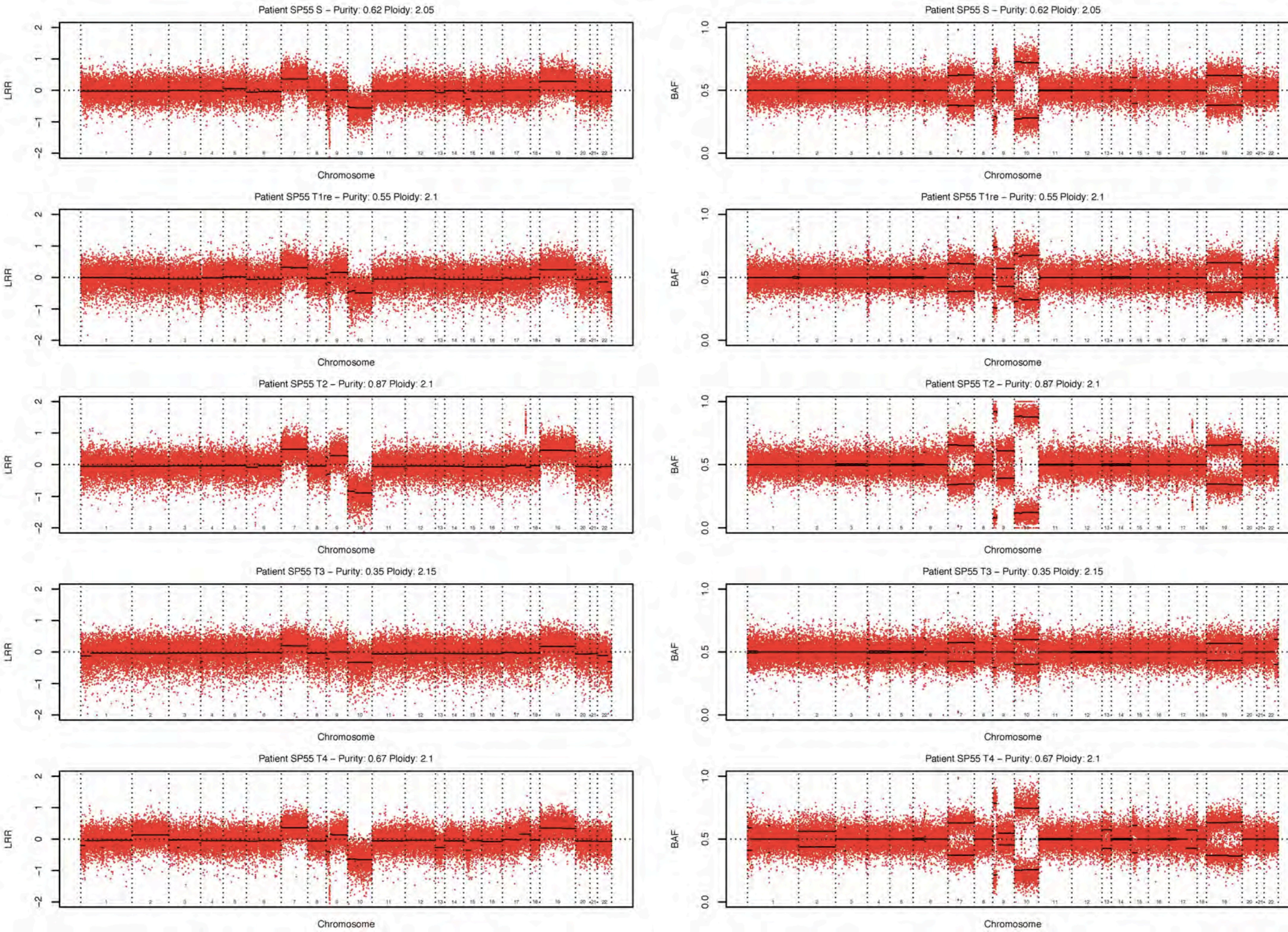

Patient SP55 S – Purity: 0.62 Ploidy: 2.05
LRR
Chromosome
Patient SP55 S – Purity: 0.62 Ploidy: 2.05
BAF
Chromosome
Patient SP55 T1re – Purity: 0.55 Ploidy: 2.1
LRR
Chromosome
Patient SP55 T1re – Purity: 0.55 Ploidy: 2.1
BAF
Chromosome
Patient SP55 T2 – Purity: 0.87 Ploidy: 2.1
LRR
Chromosome
Patient SP55 T2 – Purity: 0.87 Ploidy: 2.1
BAF
Chromosome
Patient SP55 T3 – Purity: 0.35 Ploidy: 2.15
LRR
Chromosome
Patient SP55 T3 – Purity: 0.35 Ploidy: 2.15
BAF
Chromosome
Patient SP55 T4 – Purity: 0.67 Ploidy: 2.1
LRR
Chromosome
Patient SP55 T4 – Purity: 0.67 Ploidy: 2.1
BAF
Chromosome

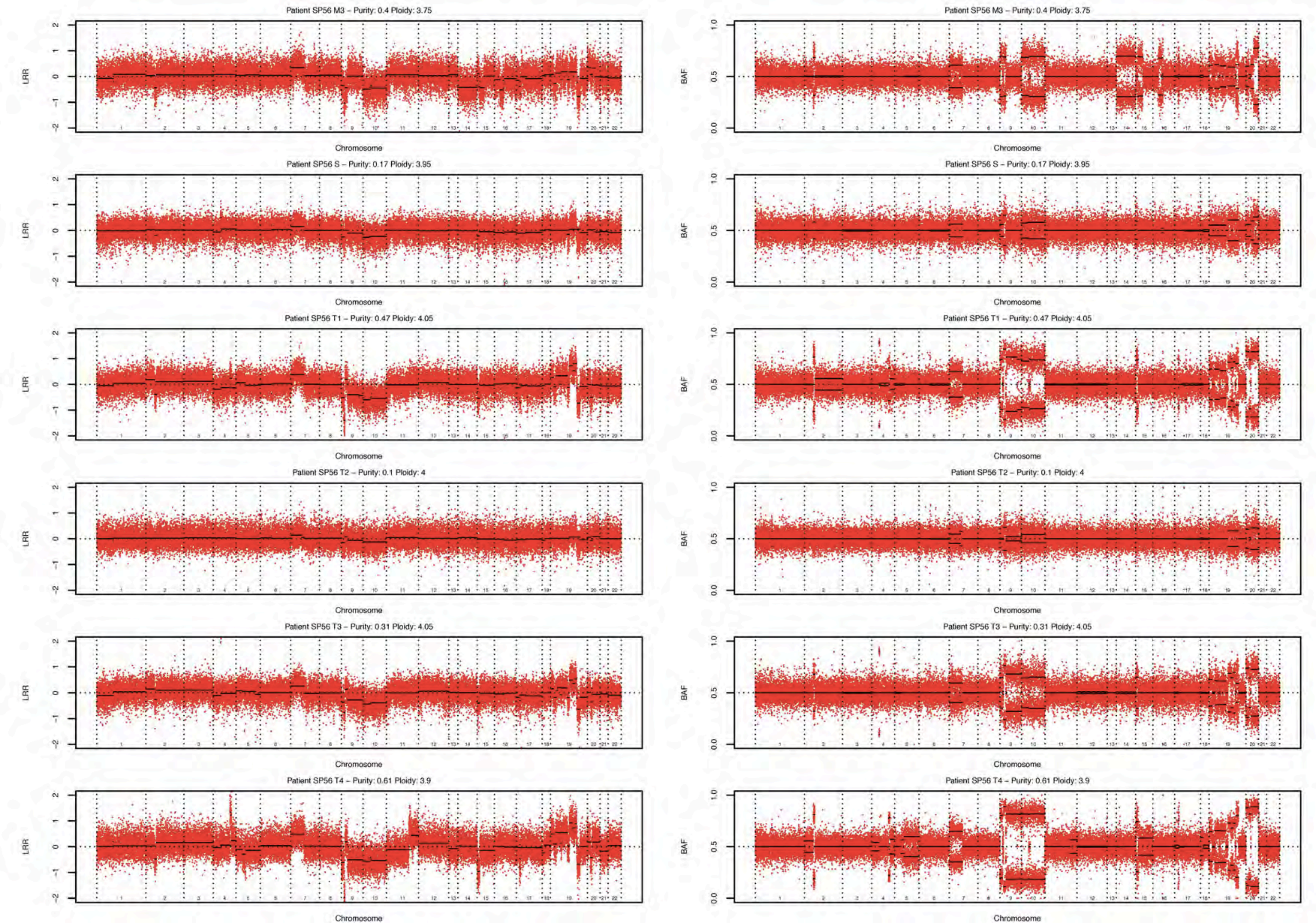
Patient SP56 M3 – Purity: 0.4 Ploidy: 3.75
LRR
Chromosome
Patient SP56 M3 – Purity: 0.4 Ploidy: 3.75
BAF
Chromosome
Patient SP56 S – Purity: 0.17 Ploidy: 3.95
LRR
Chromosome
Patient SP56 S – Purity: 0.17 Ploidy: 3.95
BAF
Chromosome
Patient SP56 T1 – Purity: 0.47 Ploidy: 4.05
LRR
Chromosome
Patient SP56 T1 – Purity: 0.47 Ploidy: 4.05
BAF
Chromosome
Patient SP56 T2 – Purity: 0.1 Ploidy: 4
LRR
Chromosome
Patient SP56 T2 – Purity: 0.1 Ploidy: 4
BAF
Chromosome
Patient SP56 T3 – Purity: 0.31 Ploidy: 4.05
LRR
Chromosome
Patient SP56 T3 – Purity: 0.31 Ploidy: 4.05
BAF
Chromosome
Patient SP56 T4 – Purity: 0.61 Ploidy: 3.9
LRR
Chromosome
Patient SP56 T4 – Purity: 0.61 Ploidy: 3.9
BAF
Chromosome

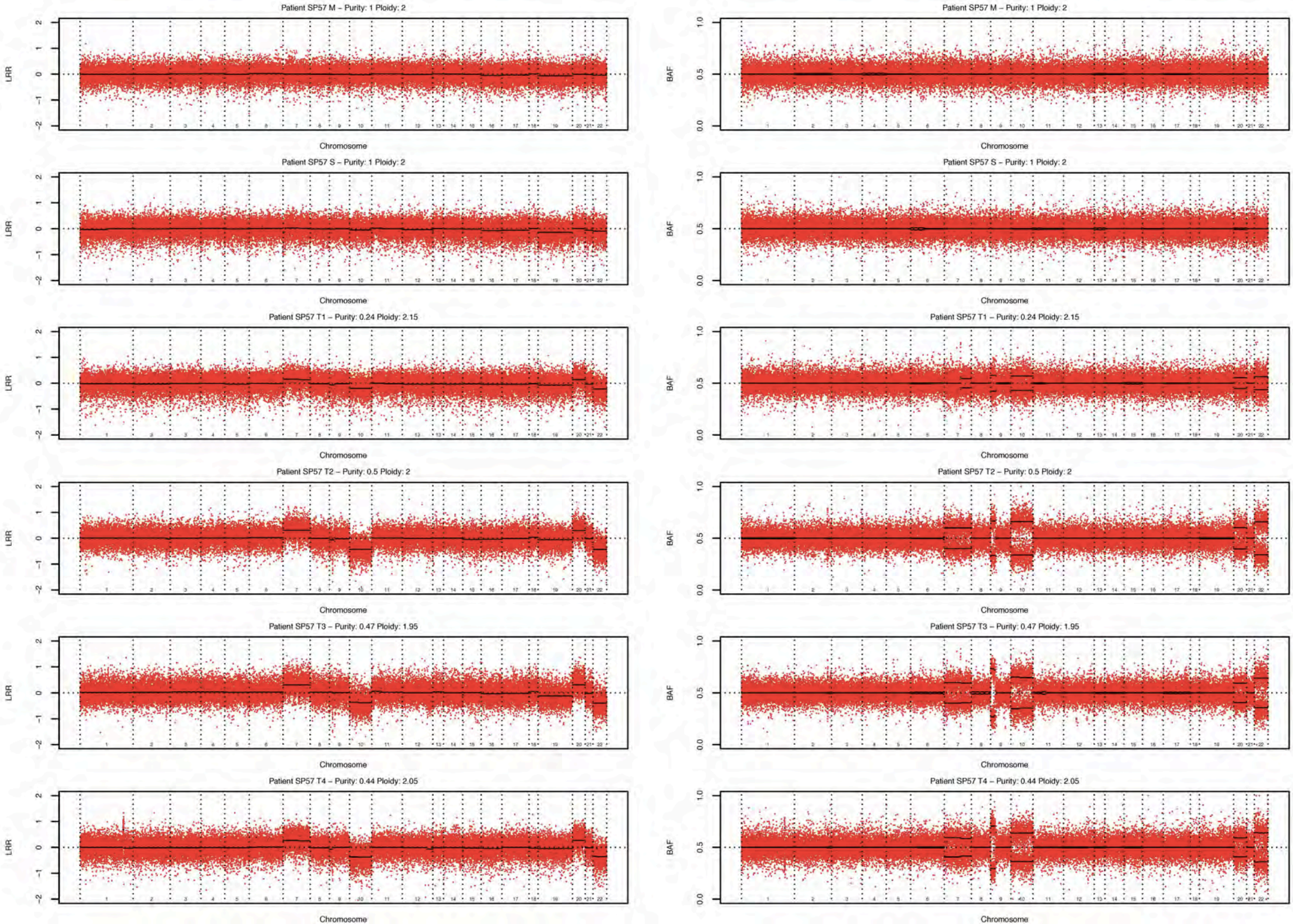
Patient SP57 M – Purity: 1 Ploidy: 2
LRR
BAF
Chromosome
Patient SP57 S – Purity: 1 Ploidy: 2
Patient SP57 T1 – Purity: 0.24 Ploidy: 2.15
Patient SP57 T2 – Purity: 0.5 Ploidy: 2
Patient SP57 T3 – Purity: 0.47 Ploidy: 1.95
Patient SP57 T4 – Purity: 0.44 Ploidy: 2.05

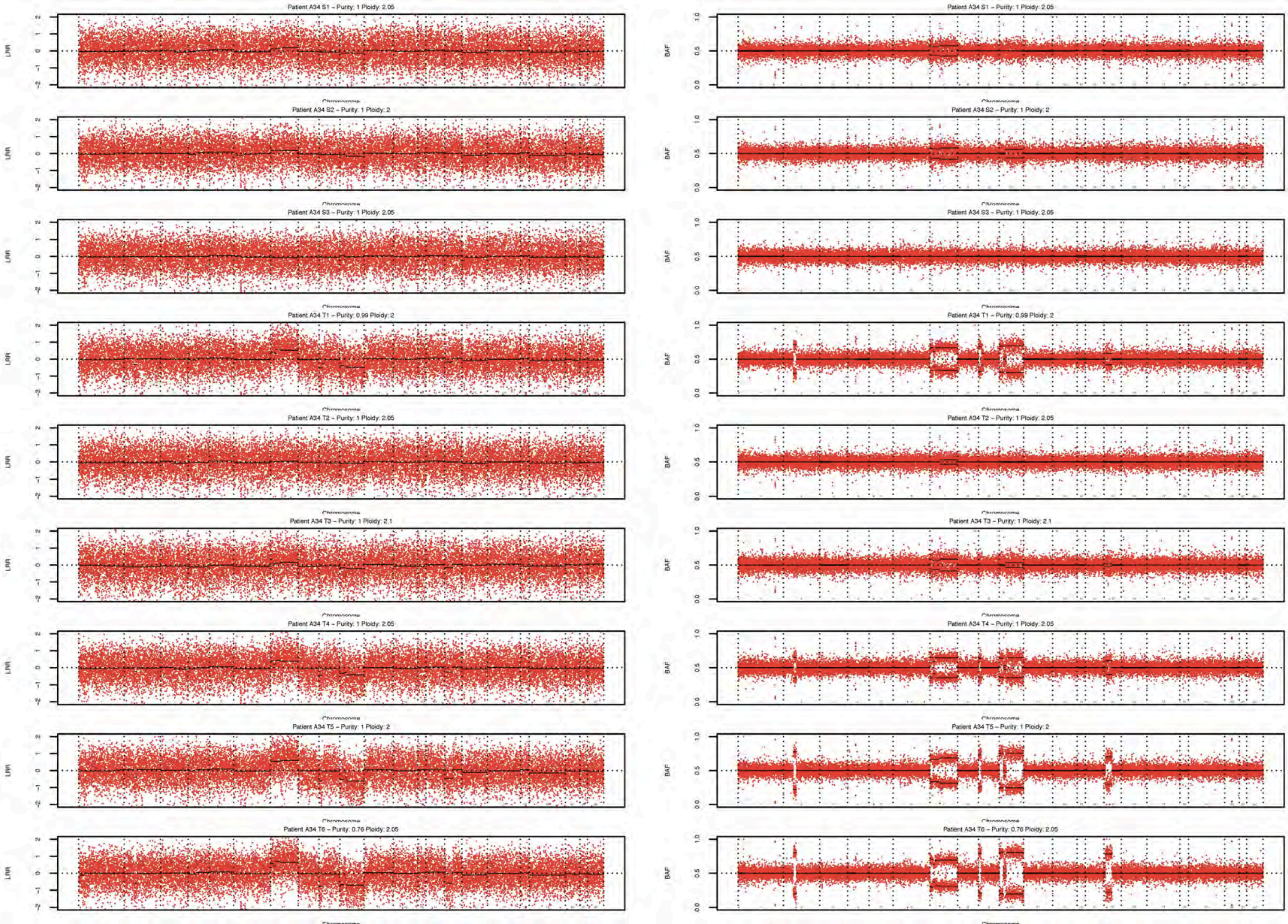
Patient A34 S1 – Purity: 1 Ploidy: 2.05
Patient A34 S2 – Purity: 1 Ploidy: 2
Patient A34 S3 – Purity: 1 Ploidy: 2.05
Patient A34 T1 – Purity: 0.99 Ploidy: 2
Patient A34 T2 – Purity: 1 Ploidy: 2.05
Patient A34 T3 – Purity: 1 Ploidy: 2.1
Patient A34 T4 – Purity: 1 Ploidy: 2.05
Patient A34 T5 – Purity: 1 Ploidy: 2
Patient A34 T6 – Purity: 0.76 Ploidy: 2.05
LRR
BAF
Chromosome

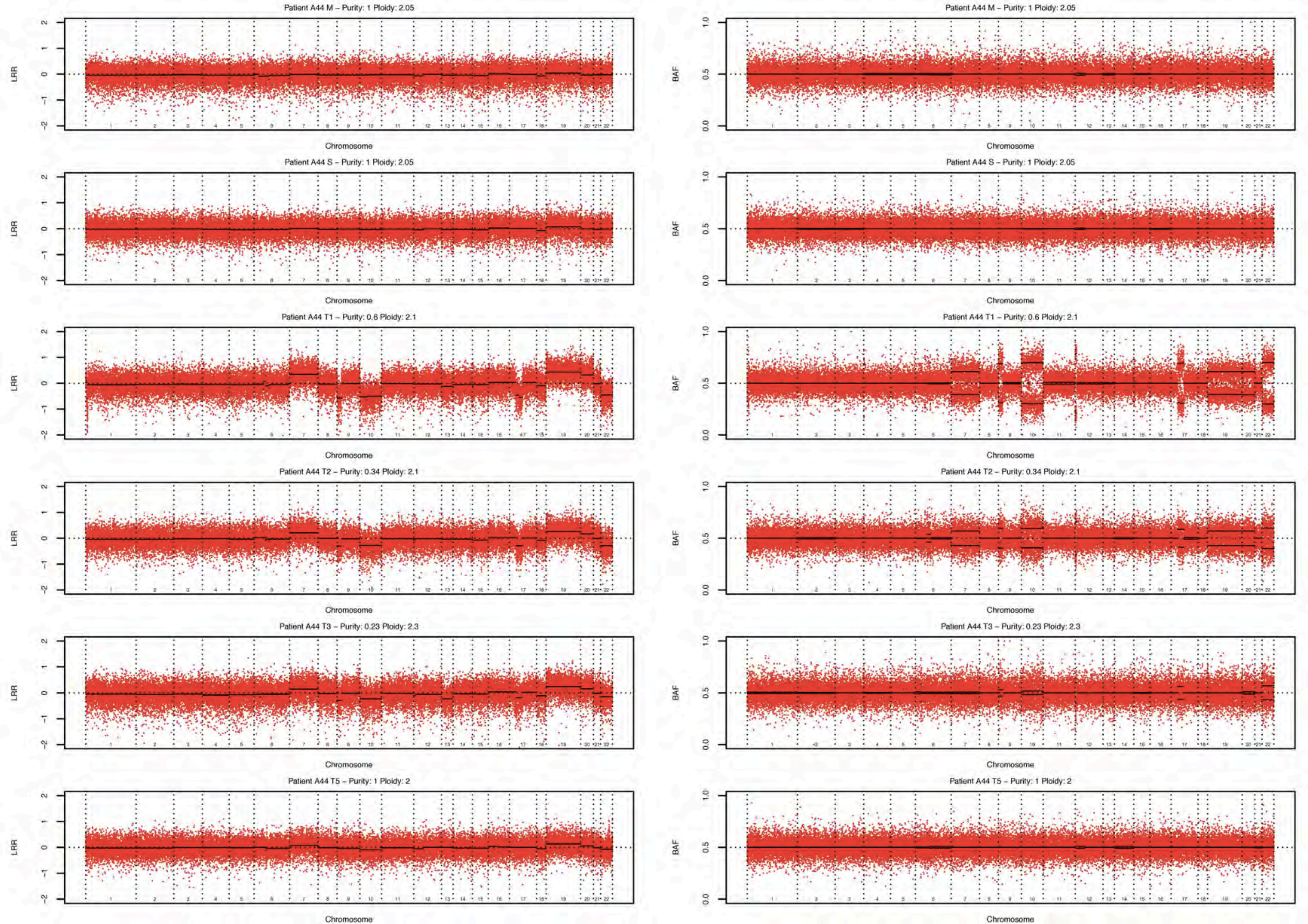
Patient A44 M – Purity: 1 Ploidy: 2.05
LRR
BAF
Chromosome
Patient A44 S – Purity: 1 Ploidy: 2.05
Patient A44 T1 – Purity: 0.6 Ploidy: 2.1
Patient A44 T2 – Purity: 0.34 Ploidy: 2.1
Patient A44 T3 – Purity: 0.23 Ploidy: 2.3
Patient A44 T5 – Purity: 1 Ploidy: 2

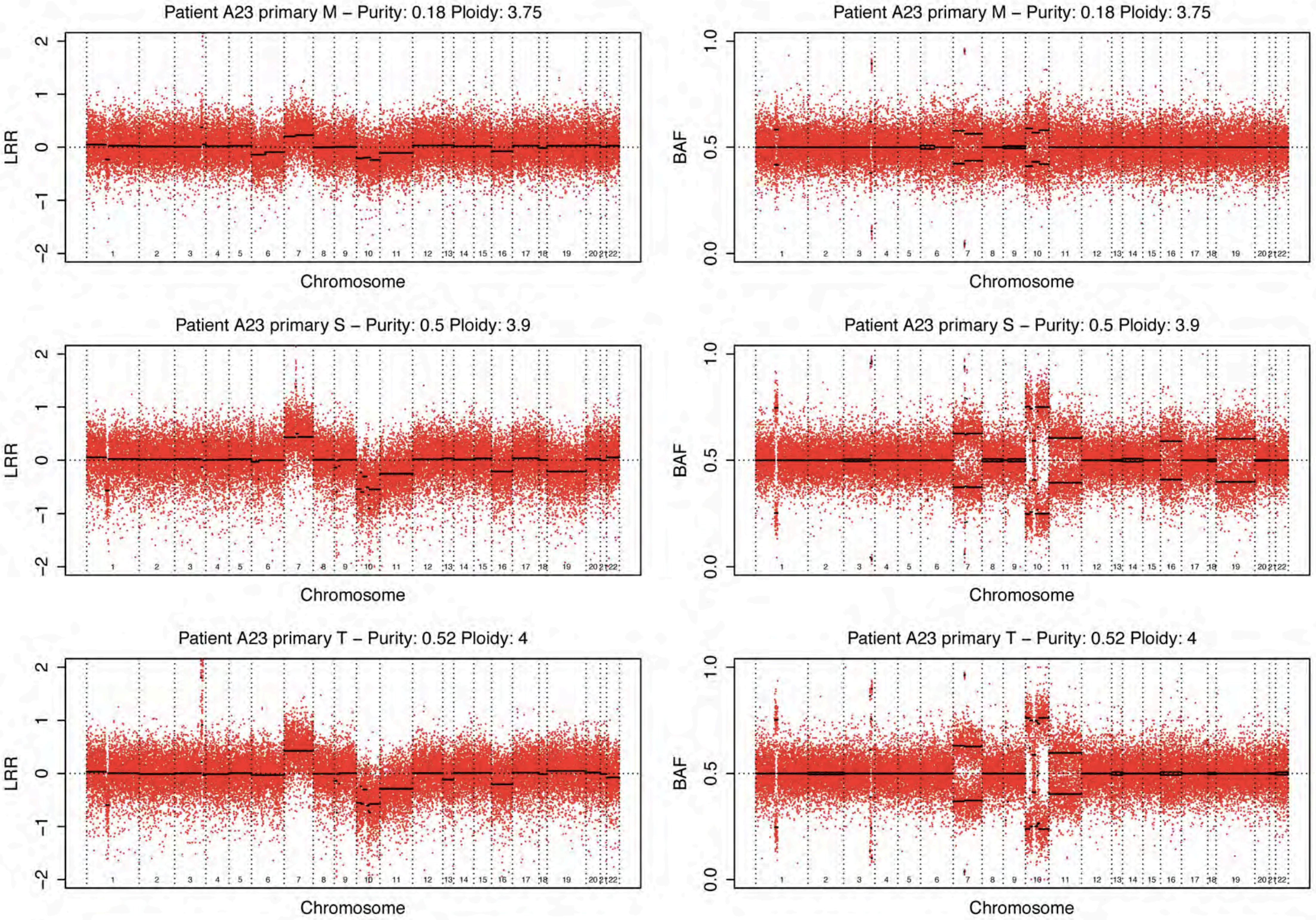
Patient A23 primary M – Purity: 0.18 Ploidy: 3.75
LRR
BAF
Chromosome
Patient A23 primary S – Purity: 0.5 Ploidy: 3.9
Patient A23 primary T – Purity: 0.52 Ploidy: 4
1 2 3 4 5 6 7 8 9 10 11 12 13 14 15 16 17 18 19 20 21 22

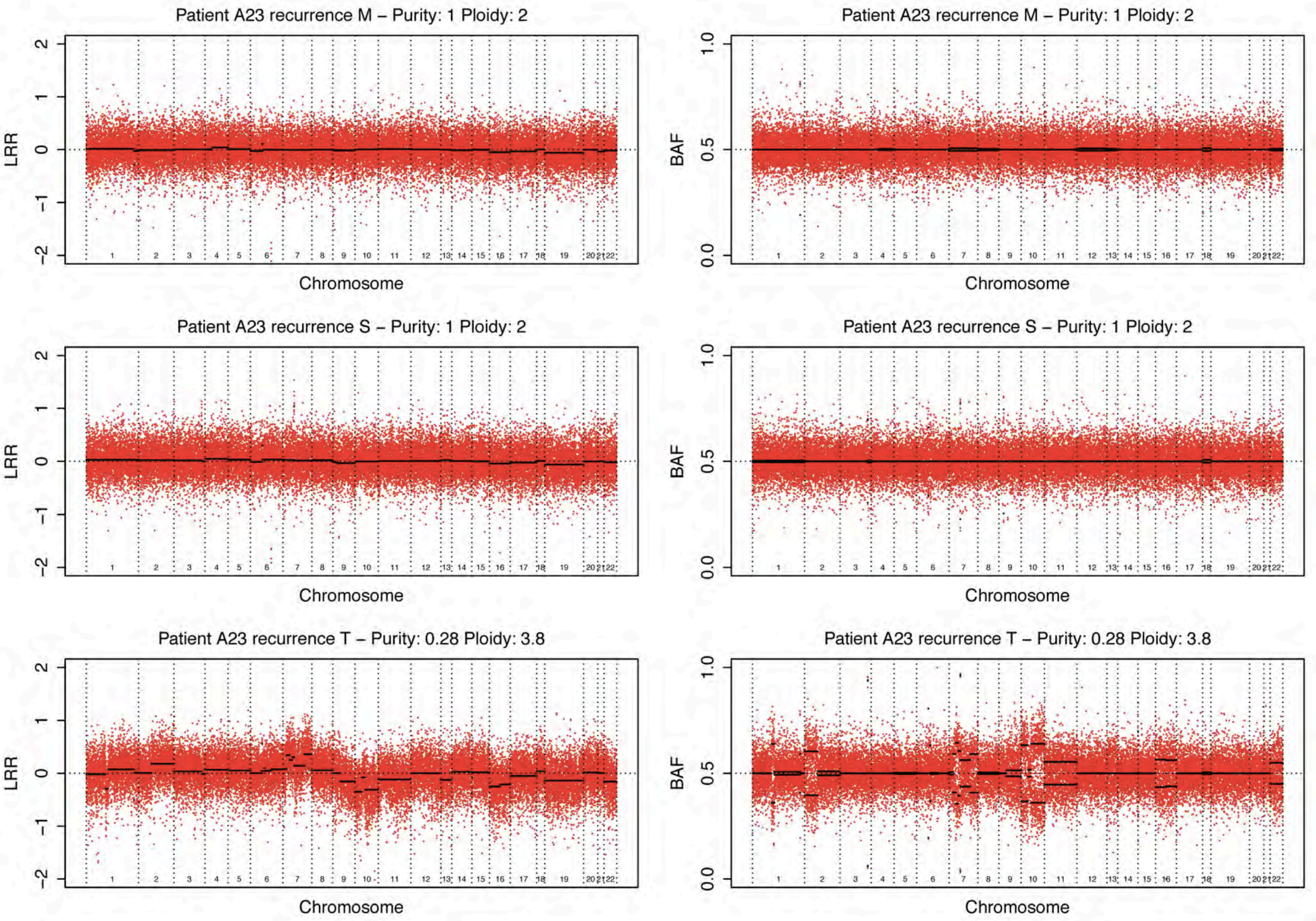
Patient A23 recurrence M – Purity: 1 Ploidy: 2
LRR
Chromosome
Patient A23 recurrence M – Purity: 1 Ploidy: 2
BAF
Chromosome
Patient A23 recurrence S – Purity: 1 Ploidy: 2
LRR
Chromosome
Patient A23 recurrence S – Purity: 1 Ploidy: 2
BAF
Chromosome
Patient A23 recurrence T – Purity: 0.28 Ploidy: 3.8
LRR
Chromosome
Patient A23 recurrence T – Purity: 0.28 Ploidy: 3.8
BAF
Chromosome

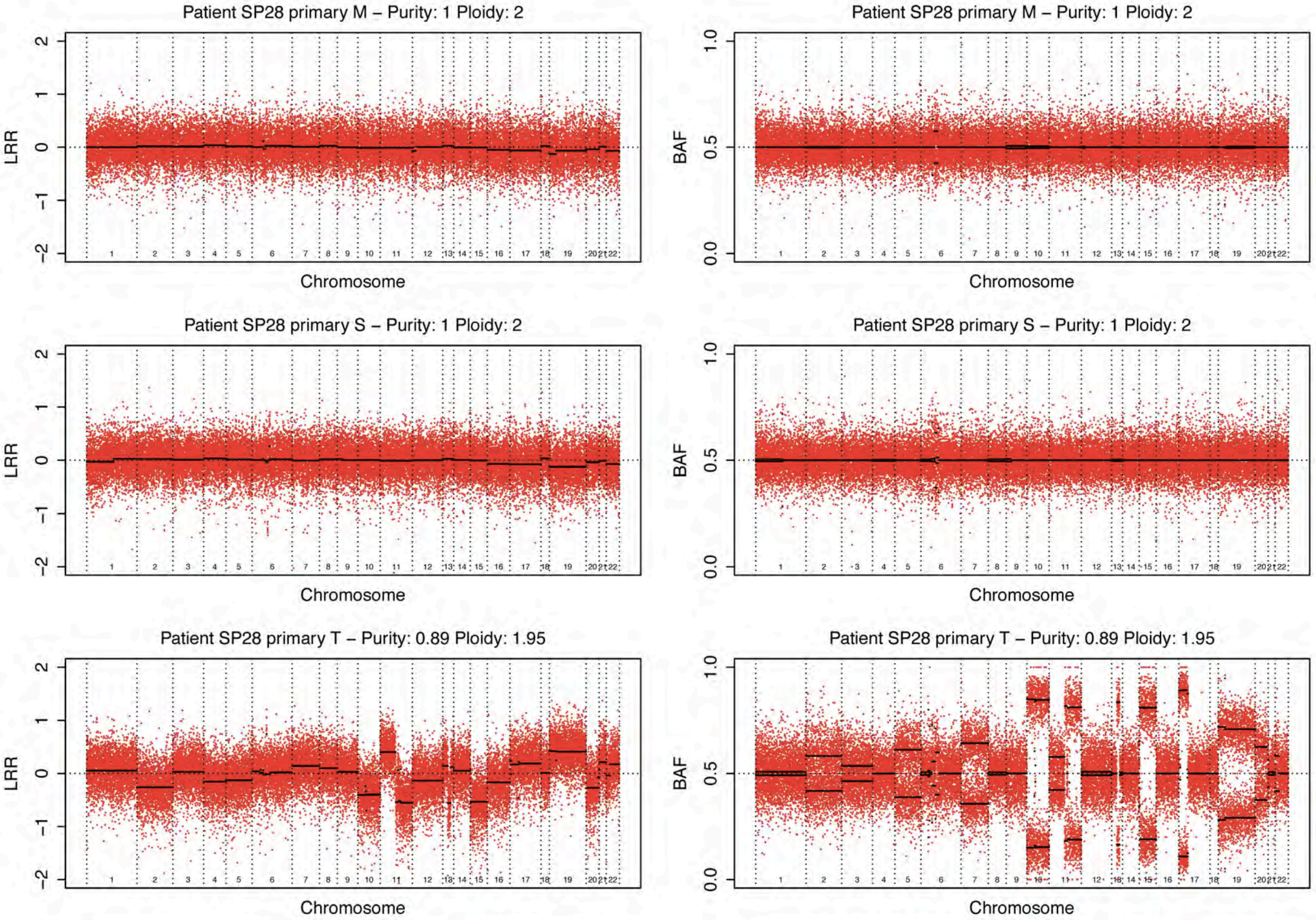
Patient SP28 primary M – Purity: 1 Ploidy: 2
LRR
Chromosome
Patient SP28 primary M – Purity: 1 Ploidy: 2
BAF
Chromosome
Patient SP28 primary S – Purity: 1 Ploidy: 2
LRR
Chromosome
Patient SP28 primary S – Purity: 1 Ploidy: 2
BAF
Chromosome
Patient SP28 primary T – Purity: 0.89 Ploidy: 1.95
LRR
Chromosome
Patient SP28 primary T – Purity: 0.89 Ploidy: 1.95
BAF
Chromosome

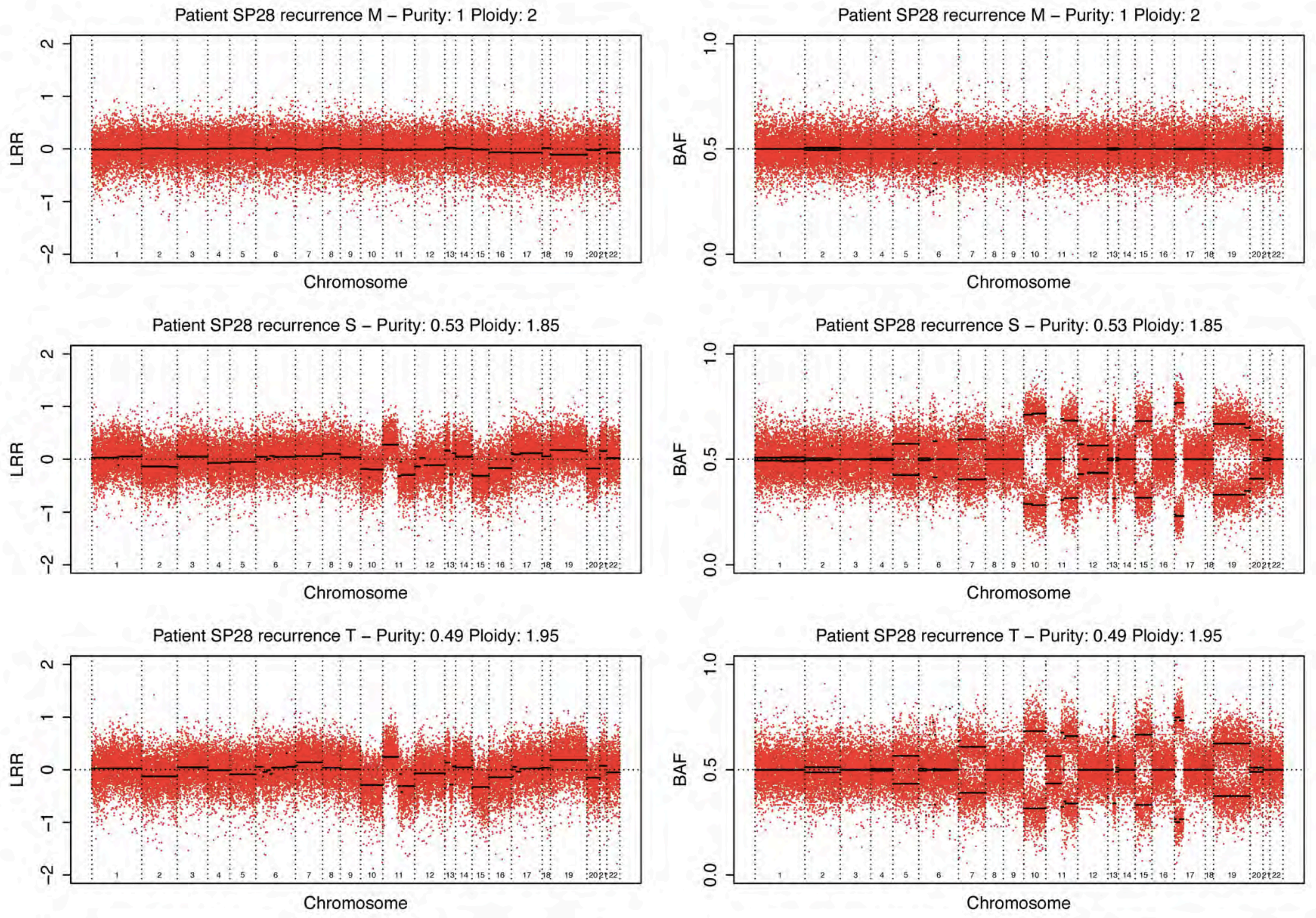
Patient SP28 recurrence M – Purity: 1 Ploidy: 2
LRR
Chromosome
BAF
Patient SP28 recurrence S – Purity: 0.53 Ploidy: 1.85
Patient SP28 recurrence T – Purity: 0.49 Ploidy: 1.95
1 2 3 4 5 6 7 8 9 10 11 12 13 14 15 16 17 18 19 20 21 22
