# Supplementary material for: Evolutionary dynamics of residual disease in human glioblastoma
Source: Ann Oncol. 2018 Nov 19;30(3):456–63. doi: 10.1093/annonc/mdy506 (PMC6442656; doi:10.1093/annonc/mdy506)
Supplement: Supplementary Data [file mdy506_supp.zip › mdy506-suppl_data/mdy506_Supplementary_Table_S1.docx]

| Sample ID | Survival after surgery (days) | Location | Histology | MIB | IDH1 | Post op radiology | F/U |
| --- | --- | --- | --- | --- | --- | --- | --- |
| SP42 | 190 | Lt parieto-occipital | GBM (Grade IV) | 52% | wt | Residual enhancement | radiotherapy |
| SP49 | 288 | Lt temporal lobe and thalamus | GBM (Grade IV) | 40% | wt | Partial resection | radiotherapy |
| SP52 | 27 | Rt Frontoparietal | GBM (Grade IV) |  | wt | Partial resection | radiotherapy |
| SP54 | 321 | Lt frontal lobe | GBM (Grade IV) | 30% | wt | Mild enhancement post op | TMZ + radiotherapy |
| SP55 | 298 | Left temporal | GBM (Grade IV) |  | wt | Residual tumour |  |
| SP56 | 86 | Rt Frontal | GBM (Grade IV) |  | wt |  | NCCU for clotting issues |
| SP57 | 354 | Rt frontal | GBM (Grade IV) |  | wt | Good resection | Concomitant and adjuvant TMZ + radiotherapy |
| A34 | alive | Lt frontal | Anaplastic astrocytoma | 20% | mut |  | Prior TMZ and radiotherapy (in 2006), re-treat with TMZ |
| A44 | 104 | Rt parietal | GBM (Grade IV) | 25% | wt | Minimal residual disease | Concomitant and adjuvant TMZ + radiotherapy |
| A23  (primary) | 1059 | Lt temporal | GBM (Grade IV) | 25% | wt | Debulking | Concomitant and adjuvant TMZ + radiotherapy |
| A23 (recurrence) | 142 | Lt temporo-parietal | GBM (Grade IV) | 31% | wt | Debulking | Rre-challenge with TMZ |
| SP28 (primary) | 588 | Lt parietal | GBM (Grade IV) |  | wt |  | Gliadel, adjuvant TMZ + radiotherapy |
| SP28 (recurrence) | 200 |  | GBM (Grade IV) |  | wt |  | For consideration of PCV chemotherapy |
