# Supplementary material for: Evolutionary dynamics of residual disease in human glioblastoma
Source: Ann Oncol. 2018 Nov 19;30(3):456–63. doi: 10.1093/annonc/mdy506 (PMC6442656; doi:10.1093/annonc/mdy506)
Supplement: Supplementary Data [file mdy506_supp.zip › mdy506-suppl_data/mdy506_Supplementary_Material.docx]

# Supplementary Material and Methods

## Bioinformatics Analysis

Purity estimation: Purity estimates were gathered from a potential clonal diploid mutation (PCDM) present in the tumour mass samples and estimation by ASCAT[1] derived from the copy number solution.

Copy number analysis: Sequenza was used to identify heterozygous single nucleotide polymorphisms (SNPs) in the WES (0.4-0.6 allele frequency in the matched normal sample) and normalise depth ratios for GC content[2]. Loci were filtered for a minimum of 25 reads in the matched normal sample. Log2 ratios (LRR) were derived from the depth ratios by calculating the log (base 2) of the depth ratio and subtracting by the global median. LRR outliers were smoothed using CGHcall[3]. The mirrored allele frequencies of the heterozygous loci were segmented using piece-wise constant fitting (PCF)[4]. If BAF values in segments are considered not to be drawn from a normal distribution expected in allele balance (BAF = 0.5, Kolmogorov-Smirnov test, p < 0.05), a two-component Gaussian mixture model was fitted to the B-allele frequency (BAF) values of the segment utilising mixtools version 1.0.4[5], in order to estimate BAFs representative of the major allele (code available as an R package at, www.github.com/georgecresswell/Mi­­­MMAl). Major allele BAF and the LRR of the genome segments were used as input for ASCAT to estimate tumour purity and ploidy, limiting the minimum purity of the solution to the lower 95% binomial confidence limit (Wilson method) of a PCDM purity estimate adjusted for a tetraploid solution. Ploidy parameter space was restricted in tumours manually determined to have high ploidy states, additionally purity and ploidy was preset if a solution was not determined. The purity and ploidy of the ASCAT solution was used to assess the clonality of each segment using the Battenberg methodology

[6]. If a segment was considered subclonal, the copy number state with the highest prevalence was taken.

Identification and validation of somatic variants: adapter trimming was performed with Skewer v0.1.126[7] specifying criteria on the length and quality of the reads (minimum of 35 after trimming and mean of 10 before trimming accordingly). Then, Burrows-Wheeler Aligner (BWA) v0.7.12[8] was used to map the reads to human reference genome hg19, and PCR duplicates were flagged using Picard tools. Mutation calling was performed in two ways: 1) with Mutect2

[9] on single tumour-normal pairs 2) with Platypus v0.8.1[10], carrying out joint mutation calling among all samples from the same patient. All mutations derived from step (1) with Mutect2 were accessioned as biased prior (‘source’) option in Platypus run, to benefit from both mutational calling methods and explore a wider range of true positive calls. Somatic variants in WES samples were filtered as follows: i) variants in segmental duplicated regions and centromeric regions were removed ii) only specific Platypus FILTER field was considered (PASS, alleleBias, Q20, QD, SC and HapScore), iii) a minimum value of 10 was accepted for coverage and genotype quality, iv) minimum of 3 reads coverage on the alternative variant in at least one of the tumour samples per patient to allow for variants in low allele frequencies to be identified, v) None of the reads covering the alternative variant should be present in the germline sample, and vi) genotype in the germline sample should be homozygous to the reference (0/0) . Mutations with a Variant Allele Frequency (VAF) <=5% were excluded. Somatic variants were annotated both with CAVA[11] and VEP[12]. SNV calling on the targeted capture samples was performed using Platypus in genotyping mode. Somatic SNVs were filtered based on i) genotype quality, minimum of 10 ii) total coverage, minimum of 300 and iii) coverage on the alternative variant, minimum of 10. Somatic mutations that failed to be validated in all samples per patient were excluded from the analysis. In any other case, VAF is indicated as NA in the failed sample. All somatic mutation calls from TES and WES panels are available in VCF files as Supplementary Data.

Driver genes: the complete set of SNVs was compared to a list of known putative driver genes in glioblastoma from ref[13].

Cancer cell fraction estimation: Cancer cell fraction of each variant is calculated using the VAF, estimated purity of the sample and total copy number state of the segment in which the variant belongs[14]. The estimated purity calculated by ASCAT was used unless the estimation was 1, then the PCDM purity estimate was used. To avoid overcalling subclonality, the number of mutated alleles was assumed to be 1.

Phylogenetic reconstruction: for each patient, SNVs identified from the whole exome sequencing panel were used to construct sample phylogenies using PAUP* (version 4.0a) with the maximum-parsimony criterion[15]. CCF values for the SNVs were first dicotomised to produce tables indicating the presence/absence of each mutation in each sample by defining a mutation as present where CCF≥0.2. For patient 54, the SVZ sample was included in the phylogenetic analysis with mutations identified by the TES2 panel and dicotomised as above. For all patients, we considered only mutations for which the presence/absence in each sample was determined. Where a mutation was absent from a margin sample (M) for a patient, but not identified as a true negative with high probability, this mutation was omitted from the phylogenetic reconstruction. The maximum-parsimony trees were identified via a heuristic search with default settings. For each phylogenetic tree, 1000 replicates of bootstrap analysis were carried out to assess the support (Figure **5**). Trees were rendered with the R package phytools[16] (R version 3.3.2, phytools version 0.6-44).

## Molecular Clock Analysis

Molecular clock phylogenetic reconstruction: FASTQ files were trimmed to remove adaptor sequences using Skewer v0.1.126 [7]. Paired reads were then aligned to hg19 using Bismark v0.18.2 [17]. Bismark methylation extractor was then used to extract methylation states of possible CpG sites from the original top and bottom strands. For each molecular clock loci called CpGs are identified. CpGs are used for analysis if they have a total number of calls for a position (methylated plus unmethylated) greater than or equal to the value at which the cumulative density function equals 0.05 for a Poisson distribution where λ is equal to the maximum CpG count in the clock region. Reads with a call missing for a genomic position that passes this coverage filter are removed to leave only complete reads with a methylation call on all locations. In each tumour sample reads are required to have at least 2 methylated CpG sites and reads can only have a maximum of 80% methylation, to remove reads that are likely produced by cells that have a low turnover (normal cells) and clocks that have reached saturation and are therefore non-informative, respectively. Remaining haplotypes (reads) with an overall abundance of 1% or less are removed due to their rarity. For each tumour sample 100 random haplotypes are selected with replacement and an additional set of 100 ‘synthetic’ unmethylated haplotypes are created as a reference for each case.

A similarity measurement is then performed pairwise between tumour samples and the unmethylated reference as used previously[18]. In brief, the Hamming distance of each haplotype combination between the two samples is measured and the shortest distance of all these combinations is recorded and the haplotype pair is removed from consideration. This is performed iteratively until all haplotype pairs have been removed and the Hamming distances of the chosen pairs is summed. This similarity measurement between all tumour samples and the unmethylated reference is used to create a Neighbour Joining tree using phangorn v2.2.0 [19]. Trees are then rendered using the same method as the somatic mutation trees.

## Method for statistical testing for mutations in the margin

Training a statistical model from read-counts: For each patient, we used its CCFs and CNA values to identify putative *clonal* SNVs in exomic regions. From Platypus VCF files, we selected only entries with:

1. A single-nucleotide mutation;
2. CCF >0.8 in *all* primary samples;
3. the *same CNA status* across all primary samples.

Mutations that do not fulfil these conditions are likely subclonal, or are not SNVs, and neglected by this analysis. Read counts for clonal SNVs are extracted from VCF files reporting Number of Variants (NV, number of reads with the alternative allele) and Reads (NR, i.e., coverage).

We corrected read counts for CNA status and tumour purity, before using them to train a statistical model for the test. Correction is carried out to estimate how many of the *observed reads* $r$ come from the actual tumour. The correction is a standard procedure

[6] which uses tumour purity $\pi$ (here estimated from WES data) and tumour copy number status *c* as follows

$$\hat{r}=\frac{c*\pi}{c*\pi+2*(1- \pi)}*r$$

Factor two in the correction is the diploid copy number status of normal cells. After correction, $\hat{r}<r$ is the number of reads, out of *r*, that are estimated coming from tumour cells. To be conservative, we assume the mutation multiplicity (number of allele copies that bear the mutation) is always 1.

With the corrected coverage value, we can fit a *Beta-Binomial* with number of trials $\hat{r}$, and successes NV. A success in this experiment is the detection of a read with the mutant allele; the overall Binomial sampling consists in the repetition of this experiment with $\hat{r}$ reads (i.e., the number of reads at a locus). The contribution of the Beta distribution is to capture uncertainty over the success probability of the experiment. Hence, this statistical model describes the probability of observing $v$ mutated at coverage $\hat{r}$ as a function of the Beta distribution $B(\alpha,\beta)$

$$\mathrm{BetaBin}(v|\hat{r};\alpha,\beta)=\frac{B(v+\alpha,\hat{r}-v+\beta)\binom{\hat{r}}{v}}{B(\alpha,\beta)}$$

This quantity is related to the so-called Variant Allele Frequency (VAF), which is defined to be $v/\hat{r}$. This compound model extends Binomial sampling with over-dispersion effects that better account for non-uniform coverage in sequencing assays [20]. Technically, it is a Binomial distribution whose parameter follows a Beta distribution with hyper-parameters $\alpha$ and $\beta$, greater than 0.

Multiple Beta-Binomial models were trained for each copy number status for the input SNVs, and each WES sample of the primary tumour. By separating read counts by copy number status, we can adjust data for every non-diploid SNV in a more precise and consistent way. To train a model at minimum acceptable quality for the next test, we discarded all combination of parameters for which we do not have, at least, 10 available SNVs. When that is the case, we removed from downstream analysis this configuration of copy number status and input sample.

To learn the model parameters $\alpha$ and $\beta$from data, we used the Maximum Likelihood fitting procedure vglm for *vector generalized linear models* that is implemented in the R package VGAM (version 1.0-5).

Testing: We identified testable SNVs from two deep-sequencing targeted panels. Because panels have ~3000x coverage, we require each SNV to have at least $k=10$ reads with the variant allele (NV$\geq10$). When that is not the case, the SNV is considered missing from the panel, and it qualifies as suitable for our test. We particularly care about the ones that are missing in the margin sample (M): if the margin was ancestral to the primary tumour, we expect it to lack some SNVs that are clonal in the primary WES samples.

If a patient has more than one margin sample (e.g. Patient A23), we require the mutation to be absent across *all* margin samples. SNVs selected in this way appear indeed clonal in the primary tumour but are missing in the margin biopsy from the targeted panels. Some patients have no testable mutations (e.g. Patient 42). The SNVs that we detected from both targeted panels are pooled together, and their number of reads (NR) from the VCF files is stored; if an SNV is detected from both panels, we sum the NR values from both panels.

Read counts from the margins are corrected as with the training set. Copy number status for mutations in the margin is the same for the training set, by design choice. Purity correction instead requires some considerations. Estimation of purity $\pi$ for margin samples is hard because of the apparent high contamination of normal cells. To be conservative, we have used a fixed, worst-case low purity estimate of $\pi=0.01$ (1% tumour, 99% normal). This value is much lower than the margin’s likely true purity and renders the test harder since the power to reject the null decreases with coverage. Thus, a conservative purity estimate leads us to rescale observed coverage to lower values (i.e., we ``throw away’’ coverage from the targeted panels).

The set of SNVs to test is divided according to the copy number status and matched to the trained models. Each group is tested independent against all models trained from the different primary regions. The null hypothesis $H_{0}$ for testing a group of SNVs is the probability of detecting NV$<k$(with $k=10$) mutated reads at the corrected coverage, given the parameters $\mu,\rho$ of the matched Beta-Binomial model

$$H_{0}:\sum_{w=1}^{k} \mathrm{BetaBin}(v=w|\hat{r};\mu,\rho)$$

The p-value is hence the probability of finding less than *k* reads with the variant allele at the designed locus with coverage $\hat{r}$, given the fact that we expect for a clonal SNV the number of reads to follow a Beta-Binomial model with parameters $\alpha,\beta$ (fit from WES). Thus, rejecting $H_{0}$ means rejecting the hypothesis that the SNV is present and clonal in the margin but just at lower frequency due to purity. Combined with phylogenetic analysis, this provides strong evidence of the ancestral relation between the margin and the primary, and that these missing SNVs are real *true negatives* in M*.* The tests are executed at confidence level $\alpha=0.05$, and corrected for multiple testing with the stringent correction possible (Family-wise Error Rate, via Bonferroni).

# Supplementary Figure and Table Legends

***Figure S1. Multi-region SNV profiling of all patients.*** *For each patient we report the cancer cell fractions (>80%) for the tumour mass samples and presence/absence of mutation in all the residual disease samples for a set of representative cases (see Table S2 for purity and Supplementary Data for SNV calls). Putative SNV driver events are annotated. WES=whole exome sequencing; TES1=targeted amplicon sequencing panel 1; TES2=targeted exome capture sequencing panel 2. T1…4=tumour mass sample; SVZ=sub ventricular zone; M=margin.*


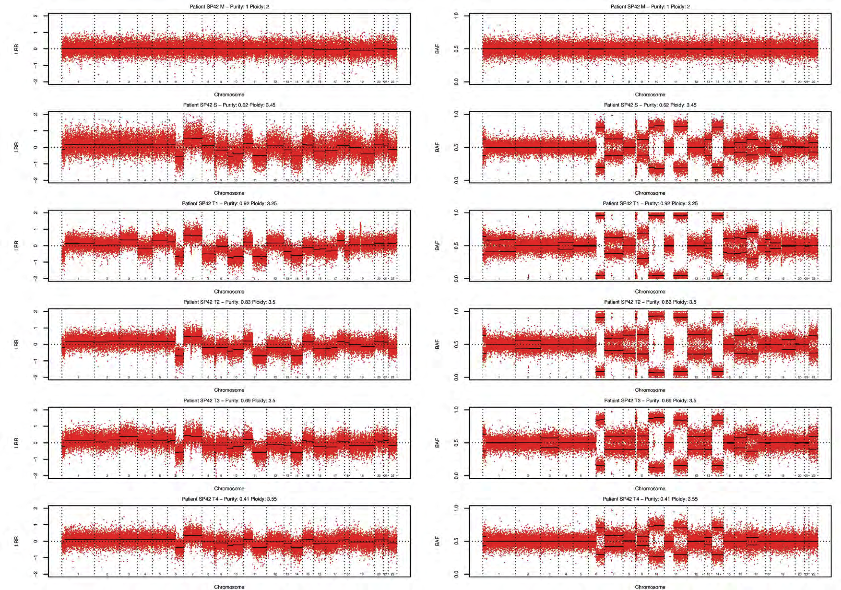
***Figure S2. Log-R-ratio and B-allele-frequency profiles per sample.*** *For each patient and sample we report the raw LRR and BAF plots based on which the digital copy number states were estimated.*


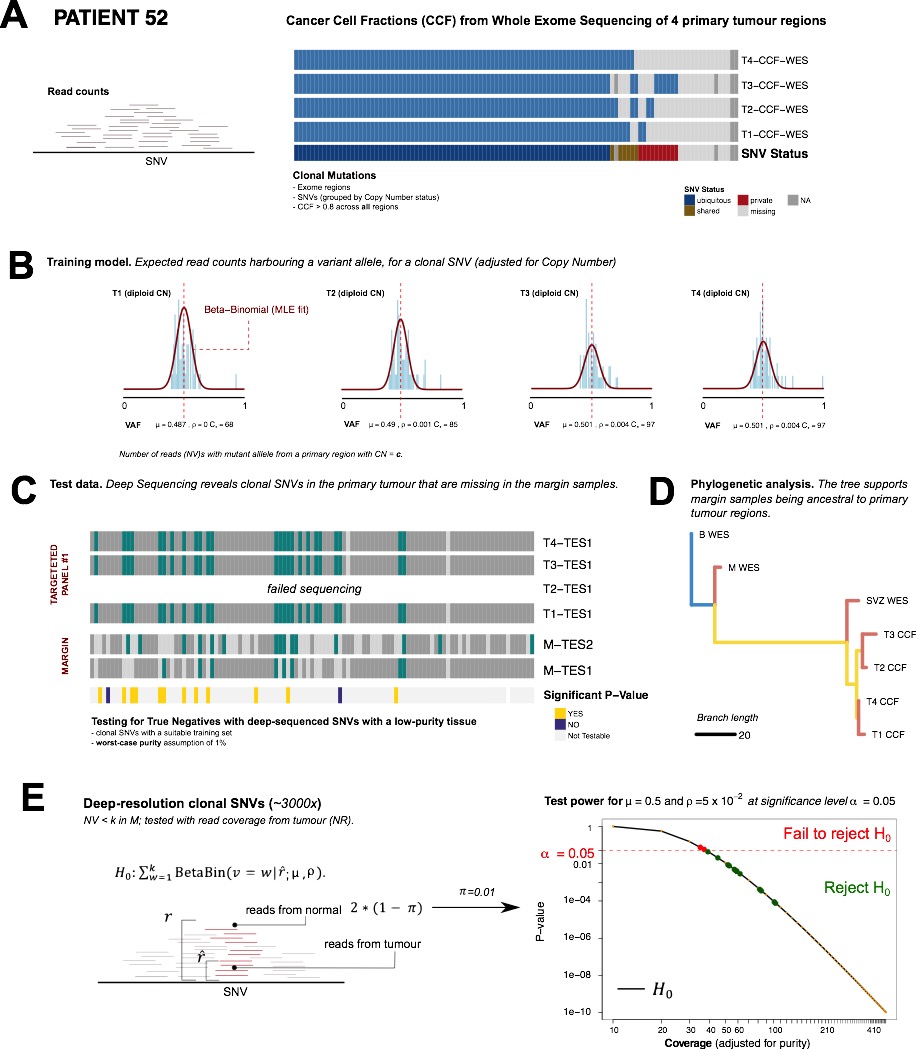


***Figure S3. Margin variant testing method. (A)*** *We analyzed read counts to estimate CCF values from WES panels of primary tumours, margins and SVZ. We group SNVs by CN status and consider clonal those with the same CN across all samples, a CCF > 0.8.* ***(B)*** *We train a Beta-Binomial model for the frequency of the mutant allele at each SNV. The training is independent for each sample (primary tumour region), and CN status.* ***(C)*** *From deep sequencing of targeted panels, we can observe that some SNVs which are clonal in the primary tumours, are missing from margin samples. We consider them missing when they have less than k=10 reads with the variant allele: this is reasonable for a deep sequencing panel.* ***(D)*** *Phylogenetic analysis of this tumour suggest that the margin could contain cells that are ancestral to the primary tumour.* ***(E)*** *We devised a test based on the Beta-Binomial models fit to the data for the null hypothesis that the missing SNVs in the margin – those that support early divergence – were not detected due to purity. With that we can compute exact p-values (adjusted for multiple comparison via Bonferroni) and isolate those where we can reject the null: those SNVs are unlikely clonal. The power of the test depends on the coverage at the analyzed SNVs. The higher the coverage, the less likely is that we have (by chance) observed k<10 mutant reads for a clonal SNV. We implement the test in very stringent condition: we assume purity of the sample 1% (very contaminated) and correct the observed coverage accordingly.*

******

***Figure S4. Phylogenetic trees bootstrapping values.*** *Phylogenetic bootstrapping values for WES trees (p=primary, r=relapse – e.g. Mr, SVZ p).*

******

***Figure S5. Phylogenetic trees using methylation clocks.*** *Phylogenetic trees reconstructed using single-allele methylation molecular clocks from a subset of analysed patients*

| Sample ID | Survival after surgery (days) | Location | Histology | MIB | IDH1 | Post op radiology | F/U |
| --- | --- | --- | --- | --- | --- | --- | --- |
| SP42 | 190 | Lt parieto-occipital | GBM (Grade IV) | 52% | wt | Residual enhancement | radiotherapy |
| SP49 | 288 | Lt temporal lobe and thalamus | GBM (Grade IV) | 40% | wt | Partial resection | radiotherapy |
| SP52 | 27 | Rt Frontoparietal | GBM (Grade IV) |  | wt | Partial resection | radiotherapy |
| SP54 | 321 | Lt frontal lobe | GBM (Grade IV) | 30% | wt | Mild enhancement post op | TMZ + radiotherapy |
| SP55 | 298 | Left temporal | GBM (Grade IV) |  | wt | Residual tumour |  |
| SP56 | 86 | Rt Frontal | GBM (Grade IV) |  | wt |  | NCCU for clotting issues |
| SP57 | 354 | Rt frontal | GBM (Grade IV) |  | wt | Good resection | Concomitant and adjuvant TMZ + radiotherapy |
| A34 | alive | Lt frontal | Anaplastic astrocytoma | 20% | mut |  | Prior TMZ and radiotherapy (in 2006), re-treat with TMZ |
| A44 | 104 | Rt parietal | GBM (Grade IV) | 25% | wt | Minimal residual disease | Concomitant and adjuvant TMZ + radiotherapy |
| A23  (primary) | 1059 | Lt temporal | GBM (Grade IV) | 25% | wt | Debulking | Concomitant and adjuvant TMZ + radiotherapy |
| A23 (recurrence) | 142 | Lt temporo-parietal | GBM (Grade IV) | 31% | wt | Debulking | Rre-challenge with TMZ |
| SP28 (primary) | 588 | Lt parietal | GBM (Grade IV) |  | wt |  | Gliadel, adjuvant TMZ + radiotherapy |
| SP28 (recurrence) | 200 |  | GBM (Grade IV) |  | wt |  | For consideration of PCV chemotherapy |

***Table S1. Clinical and follow-up information.***

***Table S2. Purity and ploidy estimates per sample.***

***Table S3. Copy number estimates per sample.***

# References

1. Van Loo P, Nordgard SH, Lingjærde OC et al. Allele-specific copy number analysis of tumors. Proc. Natl. Acad. Sci. U.S.A. 2010; 107(39):16910–16915.

2. Favero F, Joshi T, Marquard AM et al. Sequenza: allele-specific copy number and mutation profiles from tumor sequencing data. Annals of Oncology 2014; 26(1):64–70.

3. van de Wiel MA, Kim KI, Vosse SJ et al. CGHcall: calling aberrations for array CGH tumor profiles. Bioinformatics 2007; 23(7):892–894.

4. Nilsen G, Liestøl K, Van Loo P et al. Copynumber: Efficient algorithms for single- and multi-track copy number segmentation. BMC Genomics 2012 13:1 2012; 13(1):591.

5. Benaglia T, Chauveau D, Hunter DR, Young D. mixtools: An RPackage for Analyzing Finite Mixture Models. J. Stat. Soft. 2009. doi:10.18637/jss.v032.i06.

6. Nik-Zainal S, Van Loo P, Wedge DC et al. The life history of 21 breast cancers. Cell 2012; 149(5):994–1007.

7. Jiang H, Lei R, Ding S-W, Zhu S. Skewer: a fast and accurate adapter trimmer for next-generation sequencing paired-end reads. BMC Bioinformatics 2014; 15:182.

8. Li H, Durbin R. Fast and accurate short read alignment with Burrows-Wheeler transform. Bioinformatics 2009; 25(14):1754–1760.

9. Cibulskis K, Lawrence MS, Carter SL et al. Sensitive detection of somatic point mutations in impure and heterogeneous cancer samples. Nature Biotechnology 2013; 31(3):213–219.

10. Rimmer A, Phan H, Mathieson I et al. Integrating mapping-, assembly- and haplotype-based approaches for calling variants in clinical sequencing applications. Nature Genetics 2014; 46(8):912–918.

11. Münz M, Ruark E, Renwick A et al. CSN and CAVA: variant annotation tools for rapid, robust next-generation sequencing analysis in the clinical setting. Genome Med 2015; 7:76.

12. McLaren W, Gil L, Hunt SE, Riat HS. The Ensembl Variant Effect Predictor. Genome … 2016.

13. Brennan CW, Verhaak RGW, McKenna A et al. The Somatic Genomic Landscape of Glioblastoma. Cell 2013; 155(2):462–477.

14. Jiang Y, Qiu Y, Minn AJ, Zhang NR. Assessing intratumor heterogeneity and tracking longitudinal and spatial clonal evolutionary history by next-generation sequencing. PNAS 2016:201522203.

15. PAUP*: Phylogenetic Analysis Using Parsimony (and Other Methods) 4.0 Beta. [http://www.sinauer.com/paup-phylogenetic-analysis-using-parsimony-and-other-methods-4-0-beta.html].

16. Revell LJ. phytools: an R package for phylogenetic comparative biology (and other things). Methods in Ecology and Evolution 2012; 3(2):217–223.

17. Krueger F, Andrews SR. Bismark: a flexible aligner and methylation caller for Bisulfite-Seq applications. Bioinformatics 2011; 27(11):1571–1572.

18. Sottoriva A, Spiteri I, Shibata D et al. Single-molecule genomic data delineate patient-specific tumor profiles and cancer stem cell organization. Cancer Res. 2013; 73(1):41–49.

19. Schliep KP. phangorn: phylogenetic analysis in R. Bioinformatics 2011; 27(4):592–593.

20. Roth A, Khattra J, Yap D et al. PyClone: statistical inference of clonal population structure in cancer. Nat Meth 2014; 11(4):396–398.
