# Supplementary figures and images for: Evolutionary dynamics of residual disease in human glioblastoma
Source: Ann Oncol. 2018 Nov 19;30(3):456–63. doi: 10.1093/annonc/mdy506 (PMC6442656; doi:10.1093/annonc/mdy506)

***IRX2***

**A34**

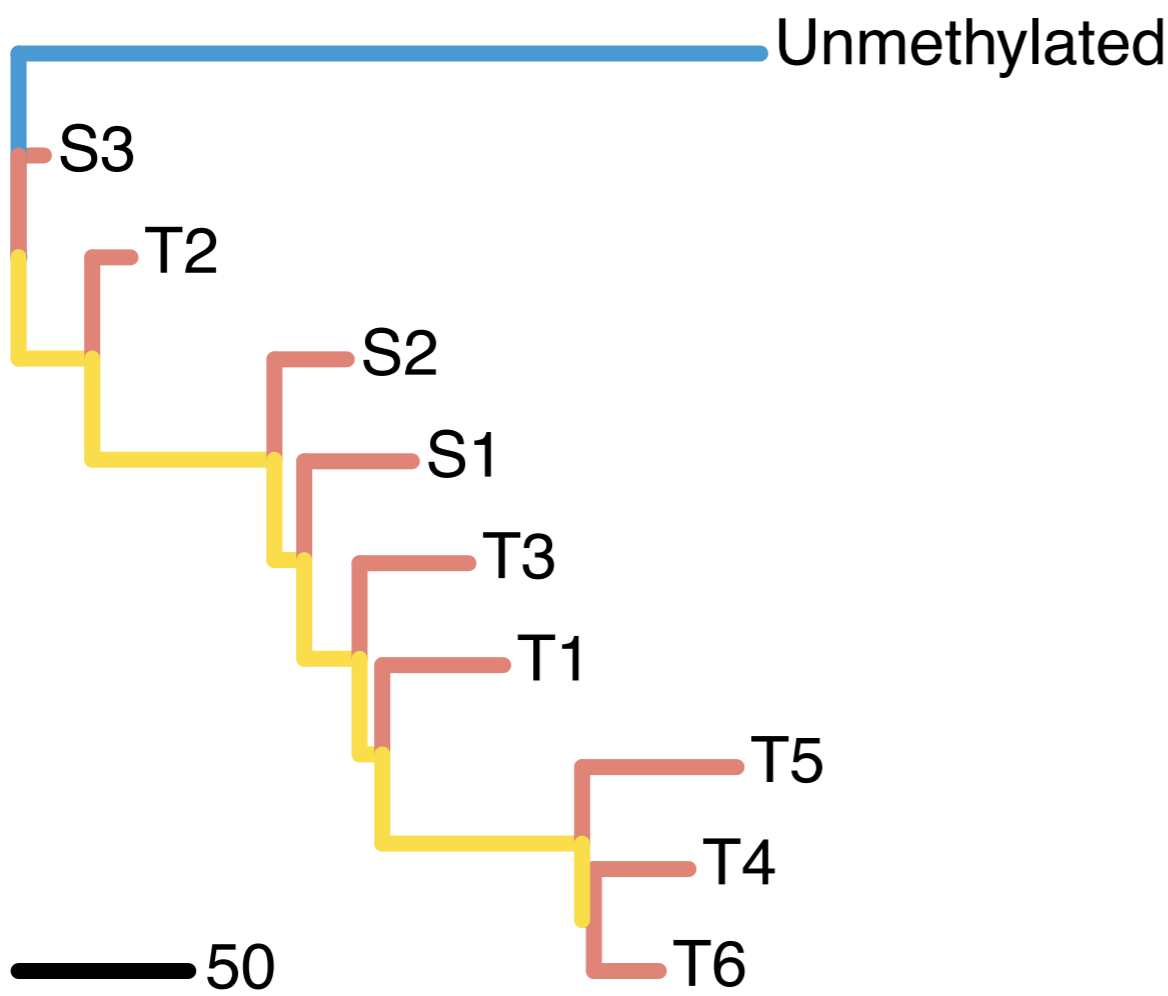

***NETO1***

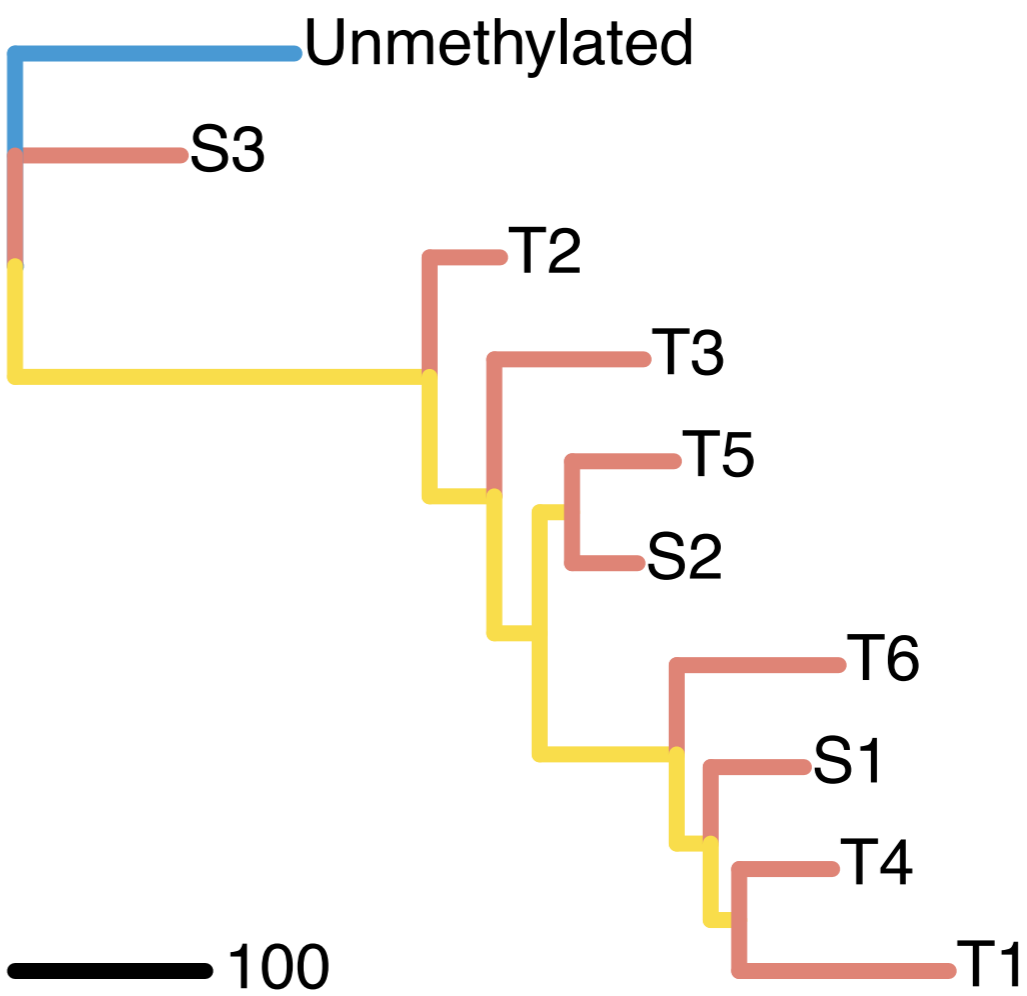

**A44**

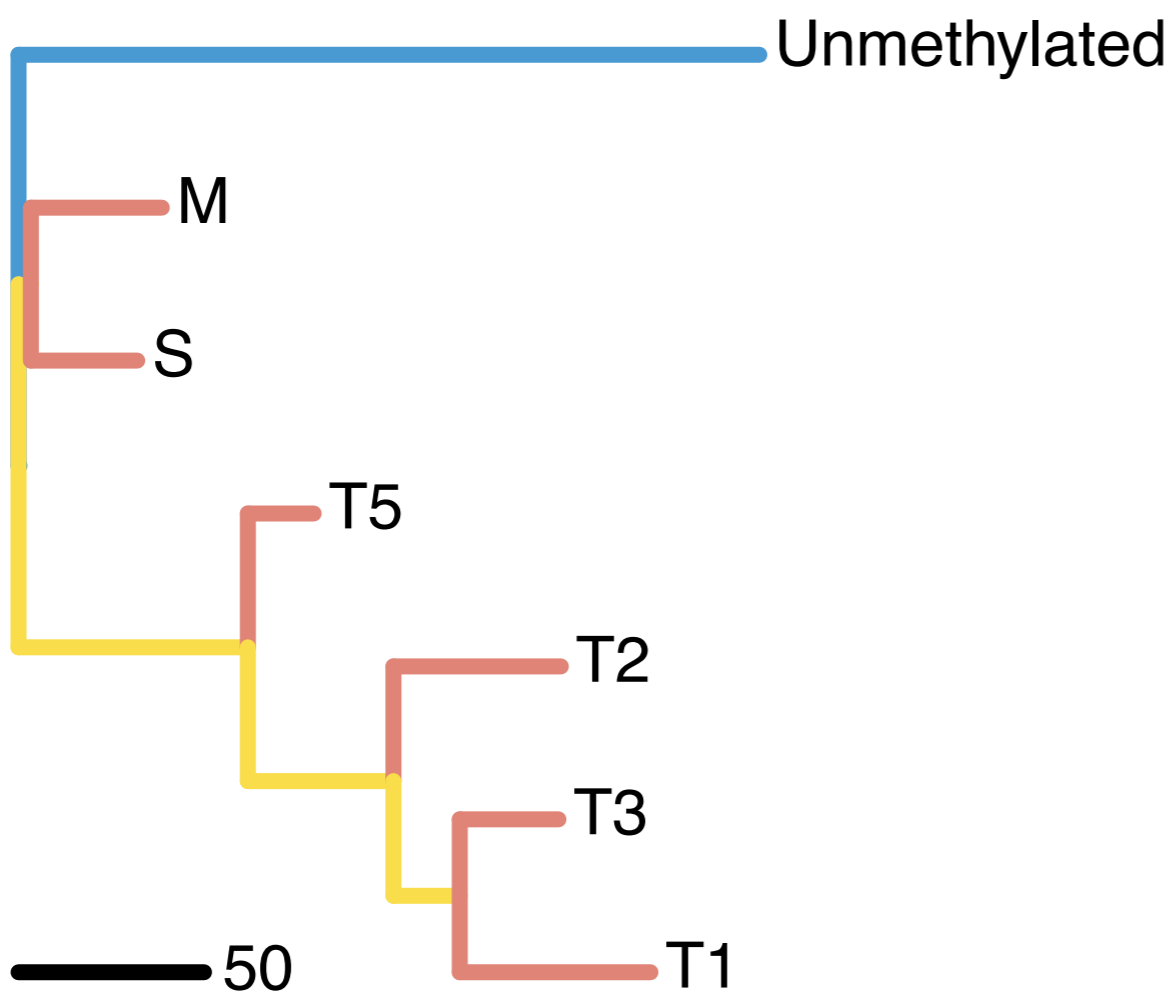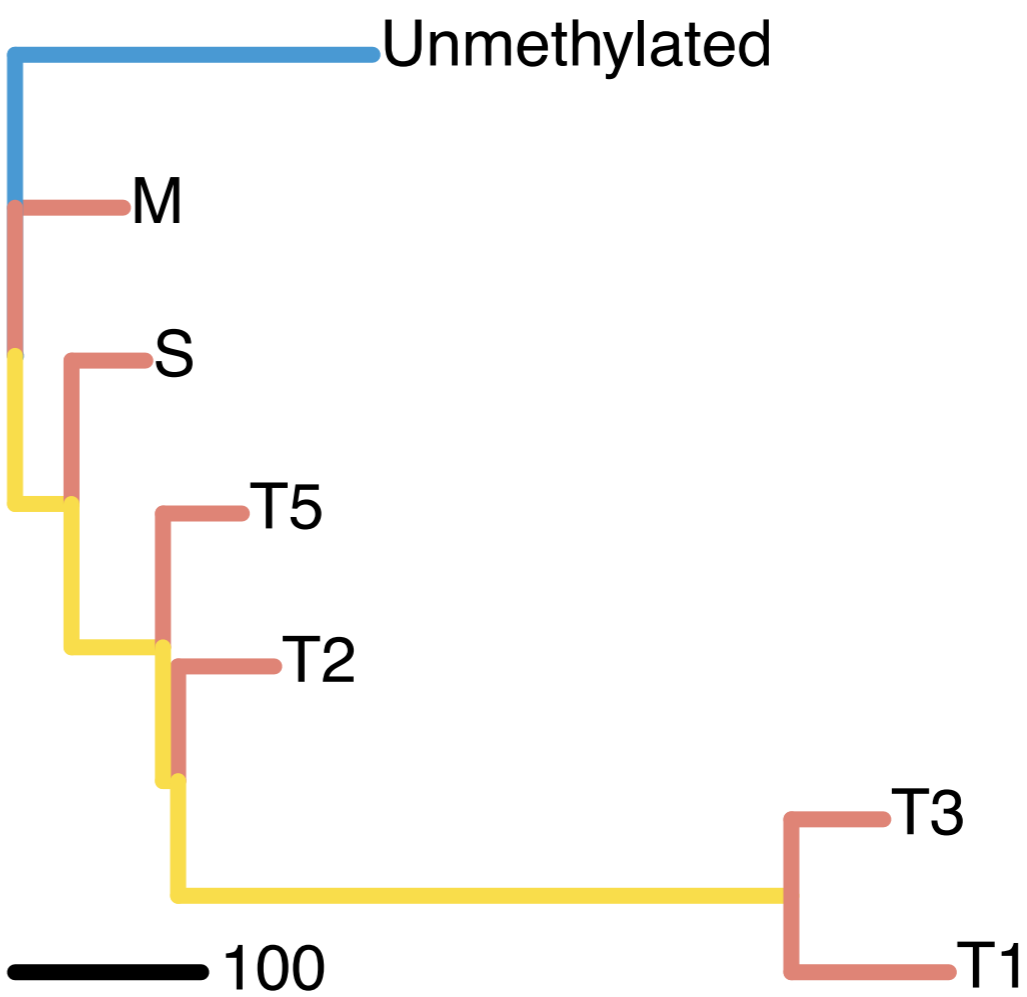

**A23**

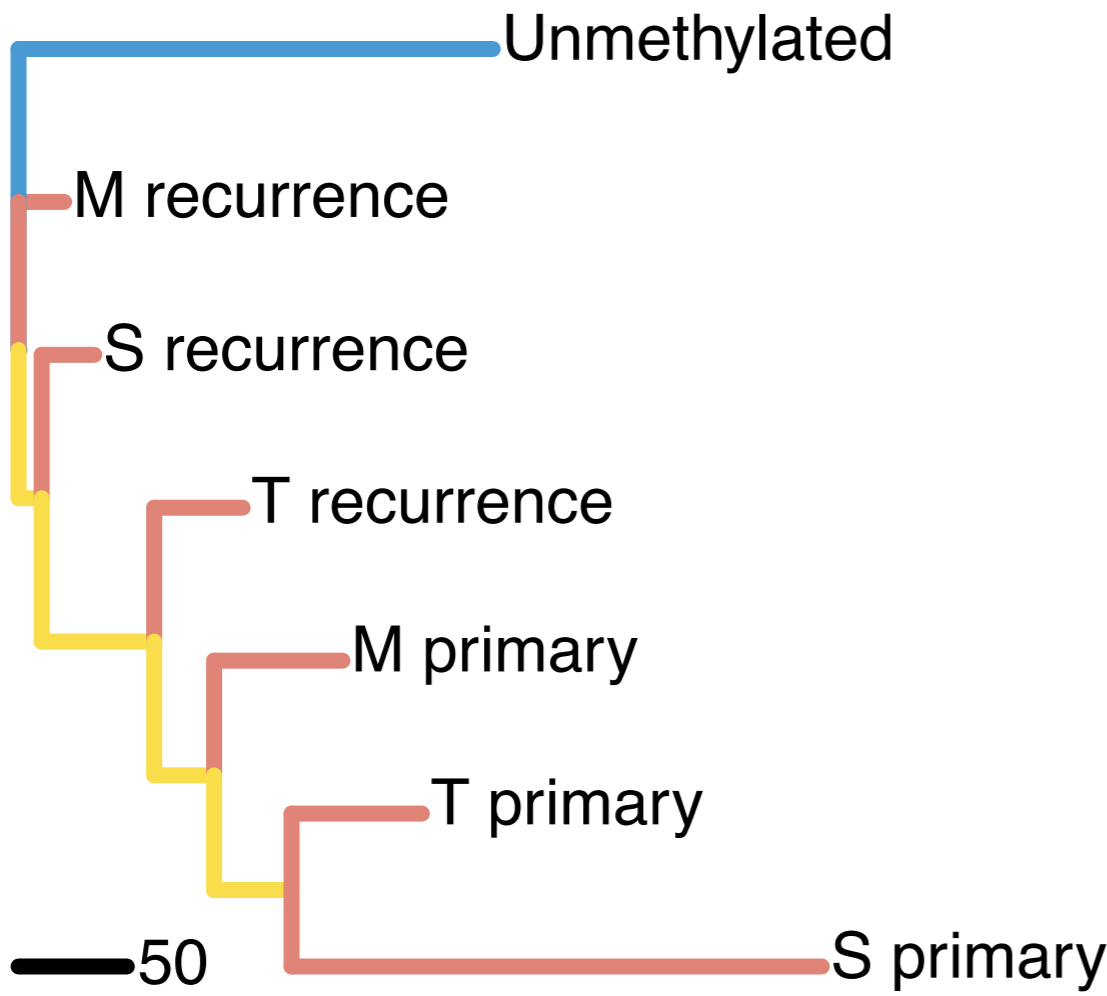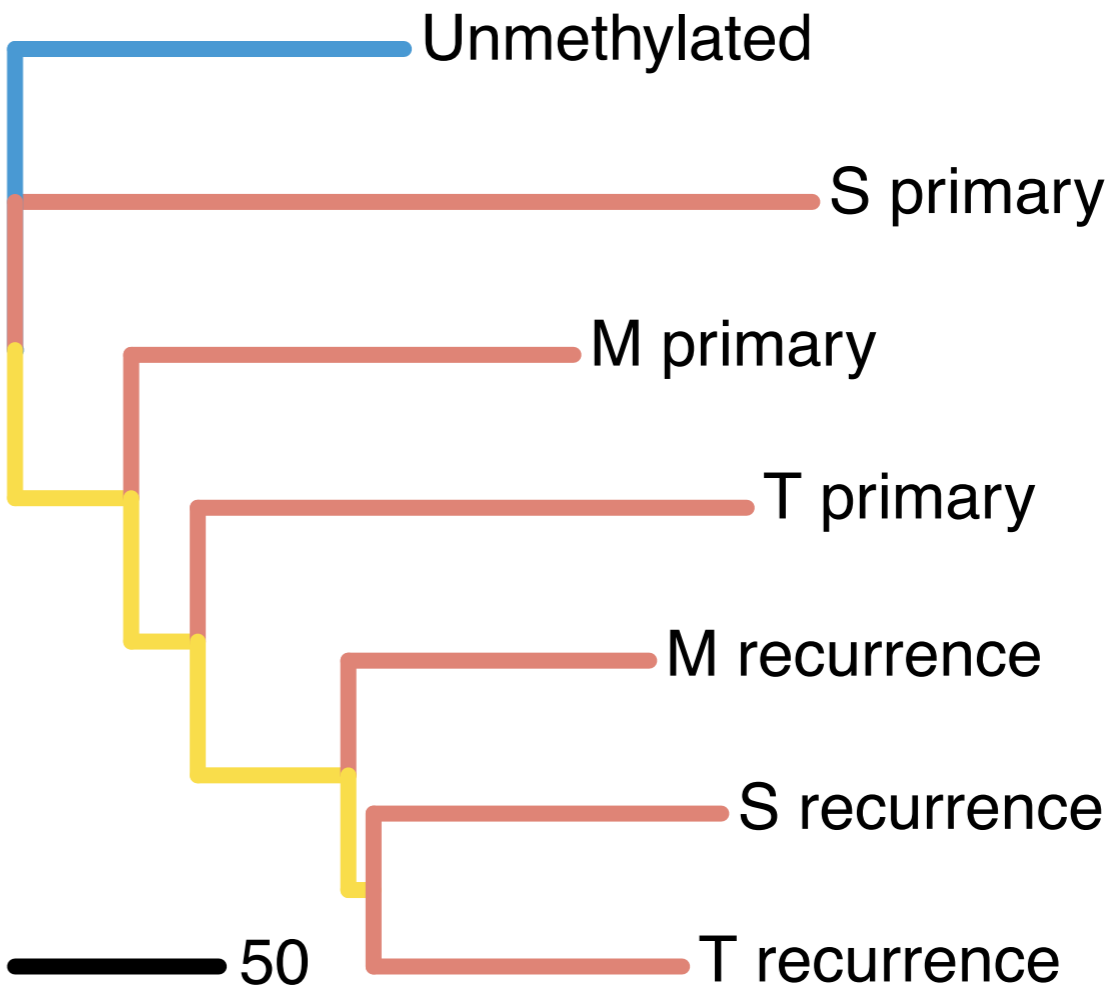

**SP28**

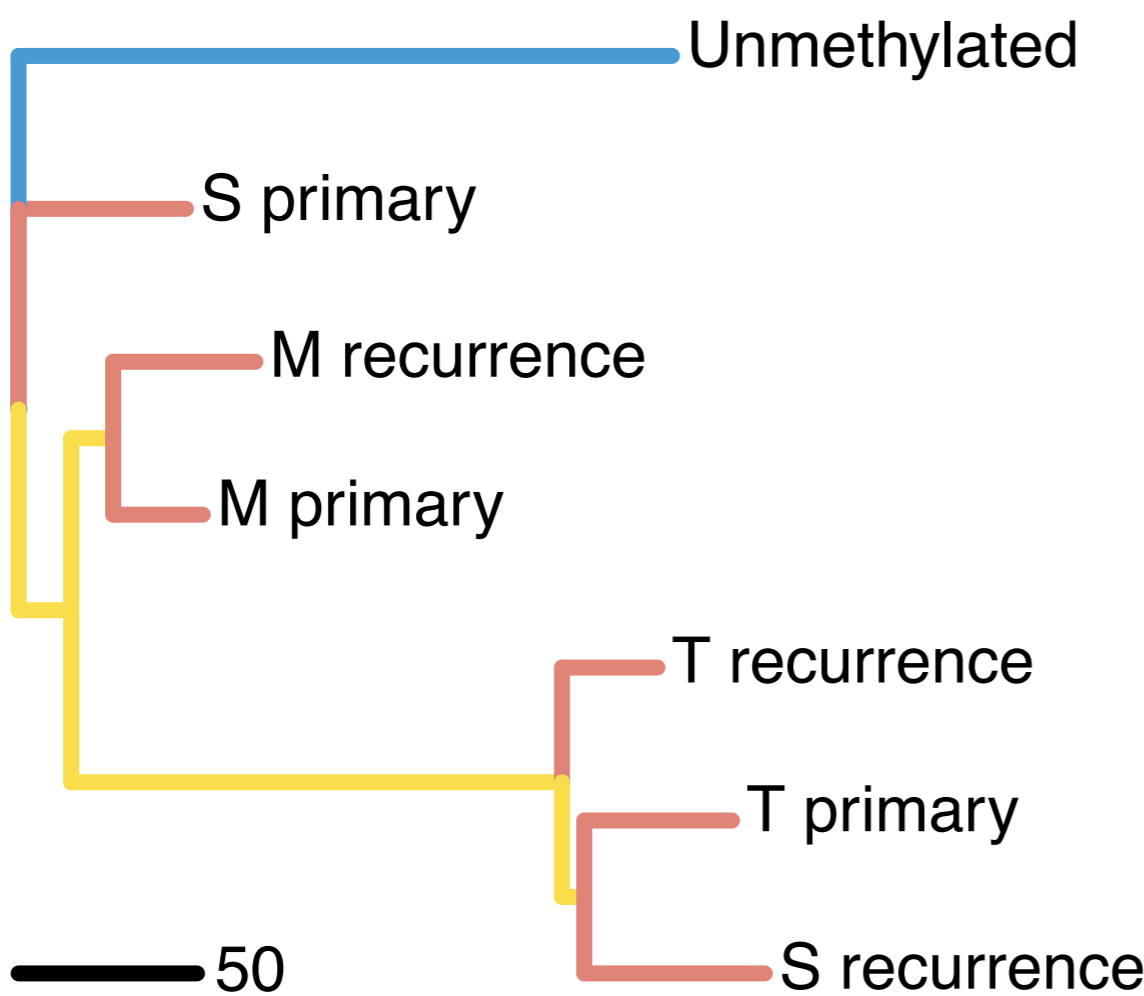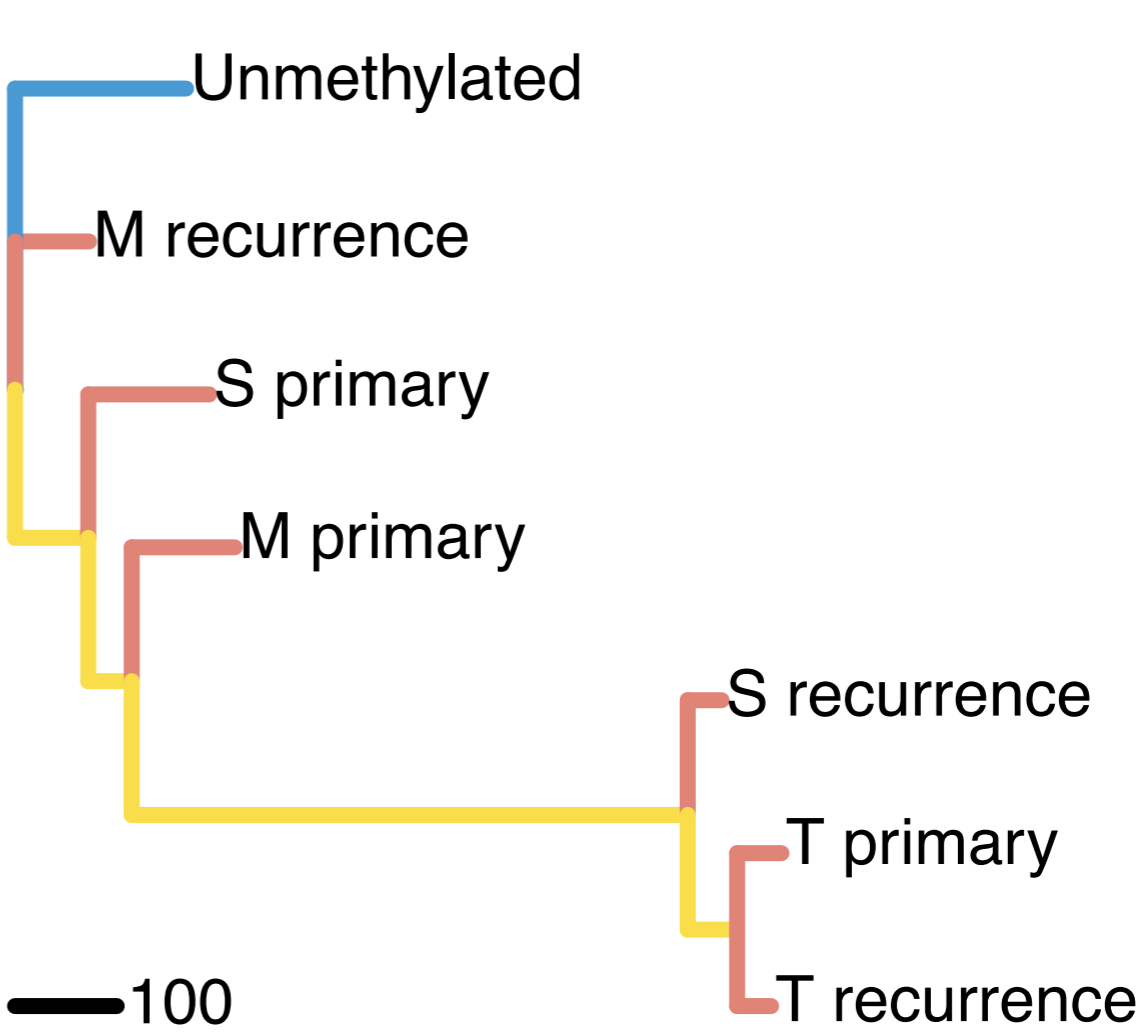

Supplement: Supplementary Data [file mdy506_supp.zip › mdy506-suppl_data/mdy506_Supplementary_Fig_S5.pdf]

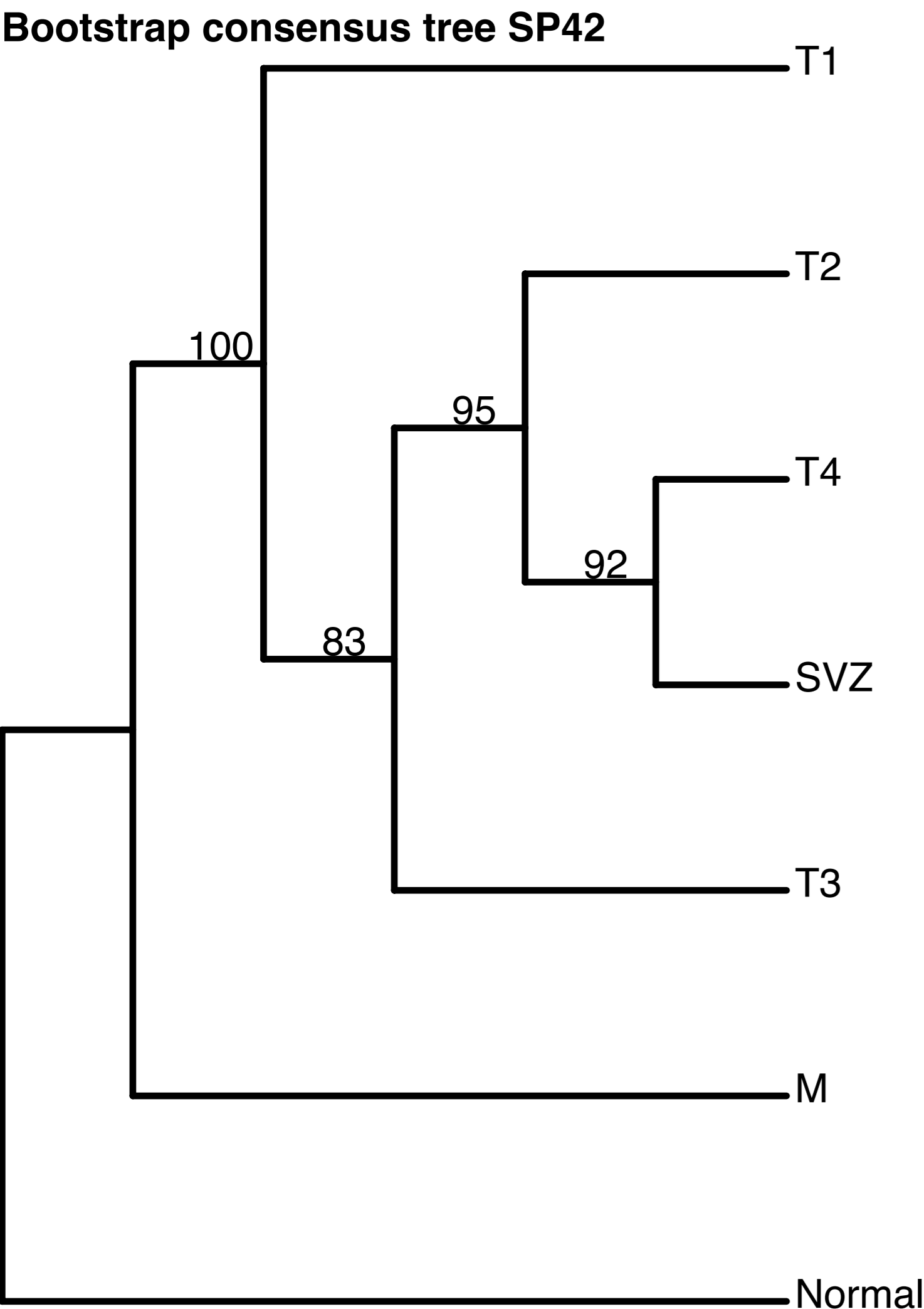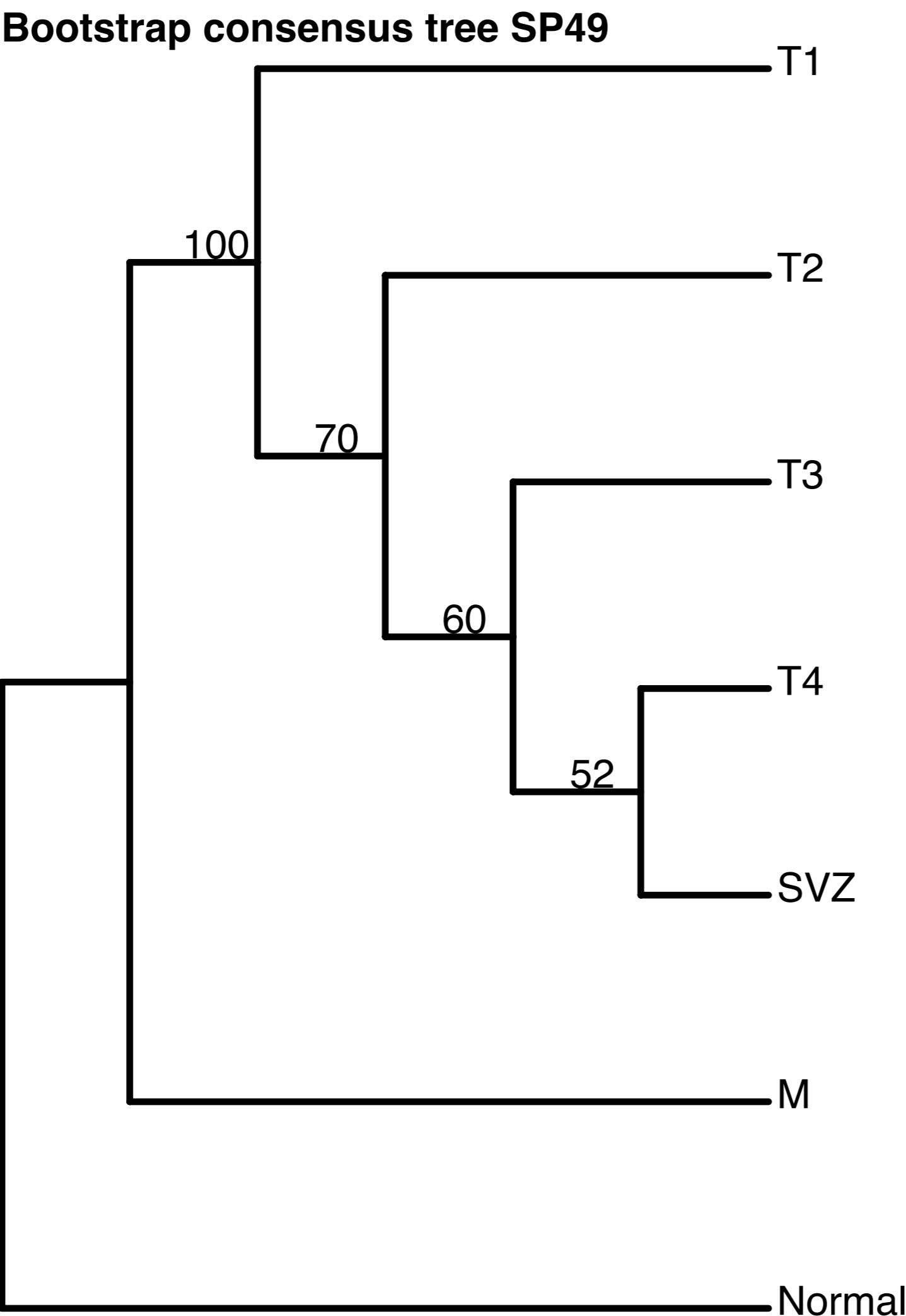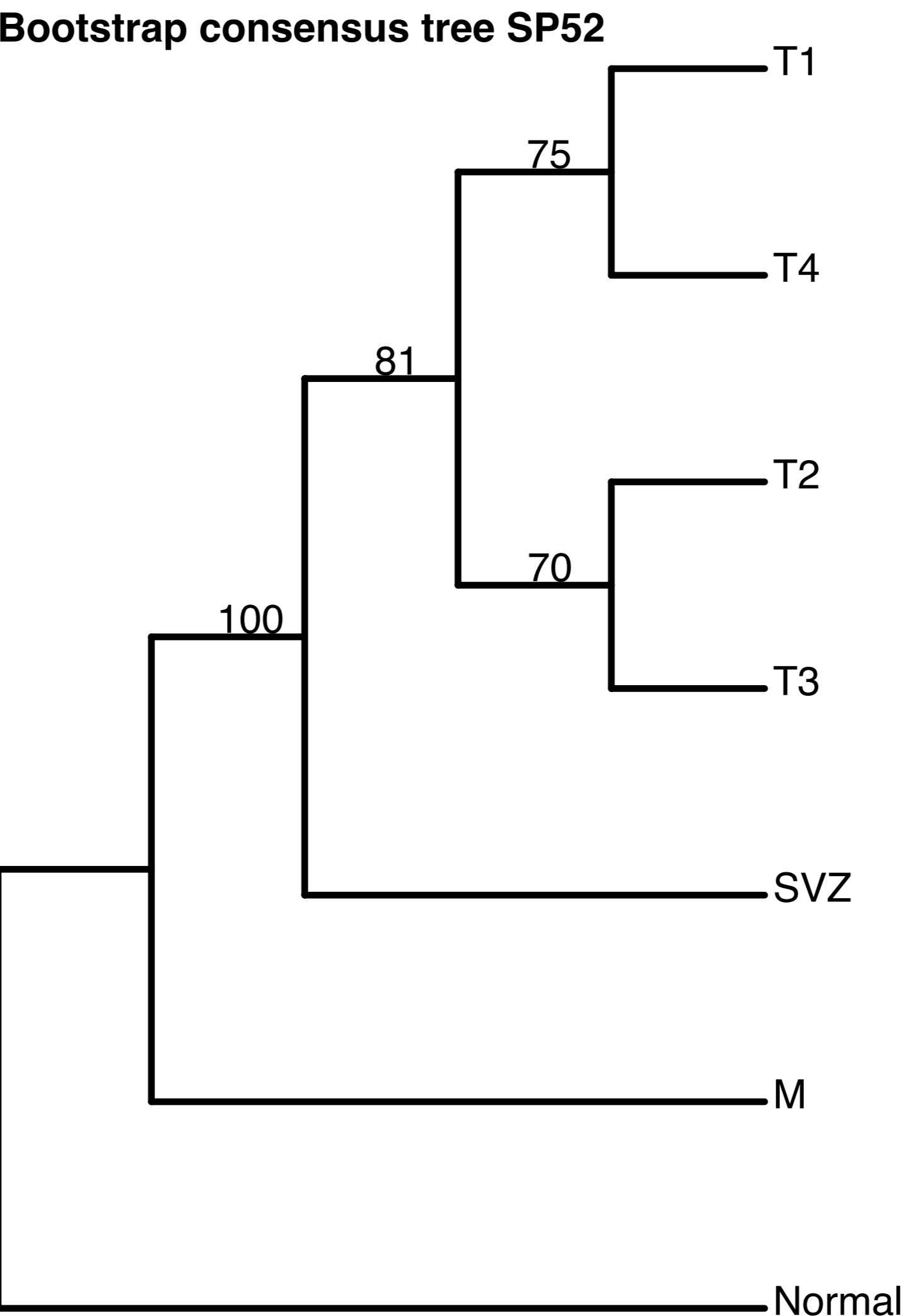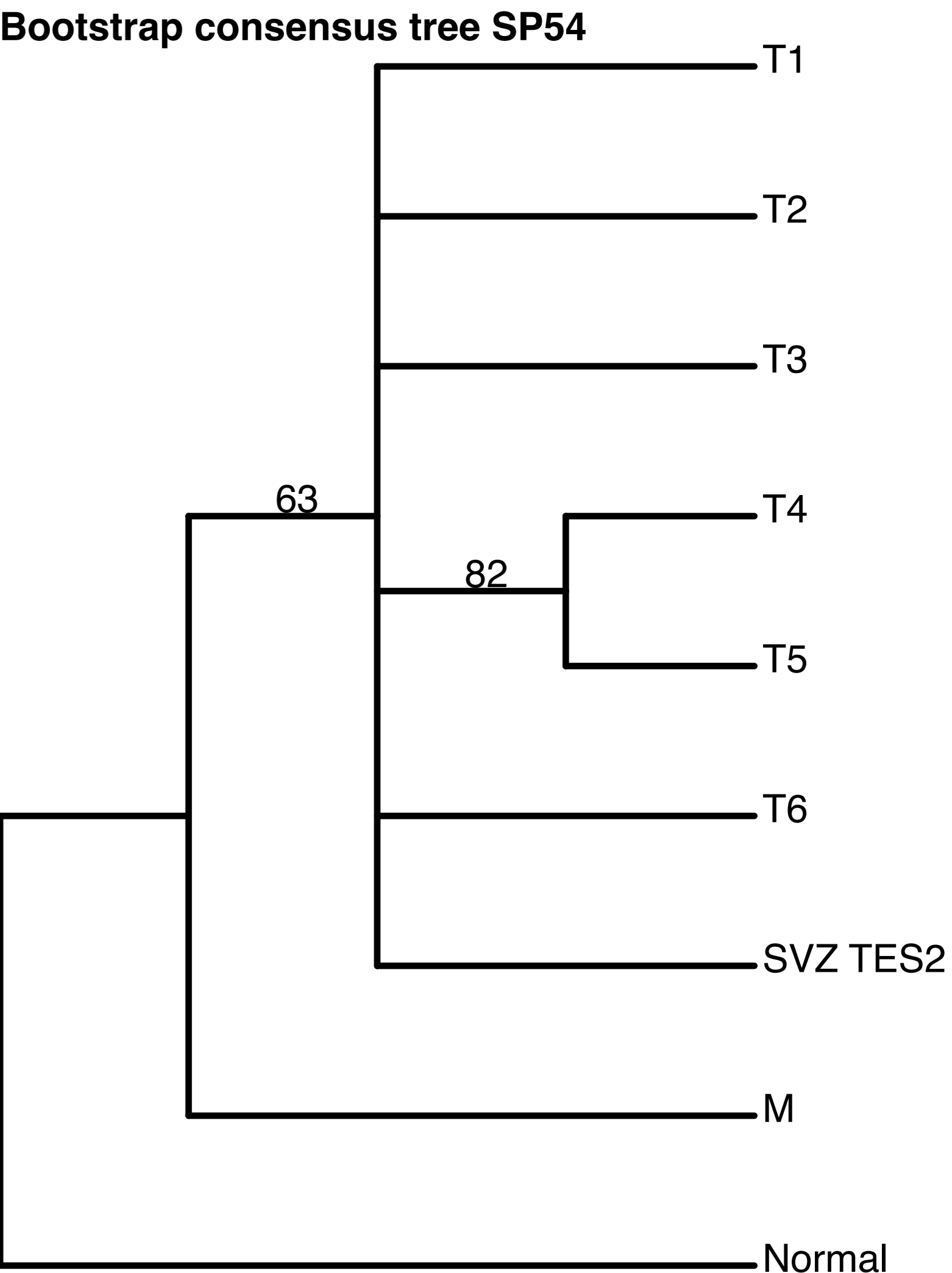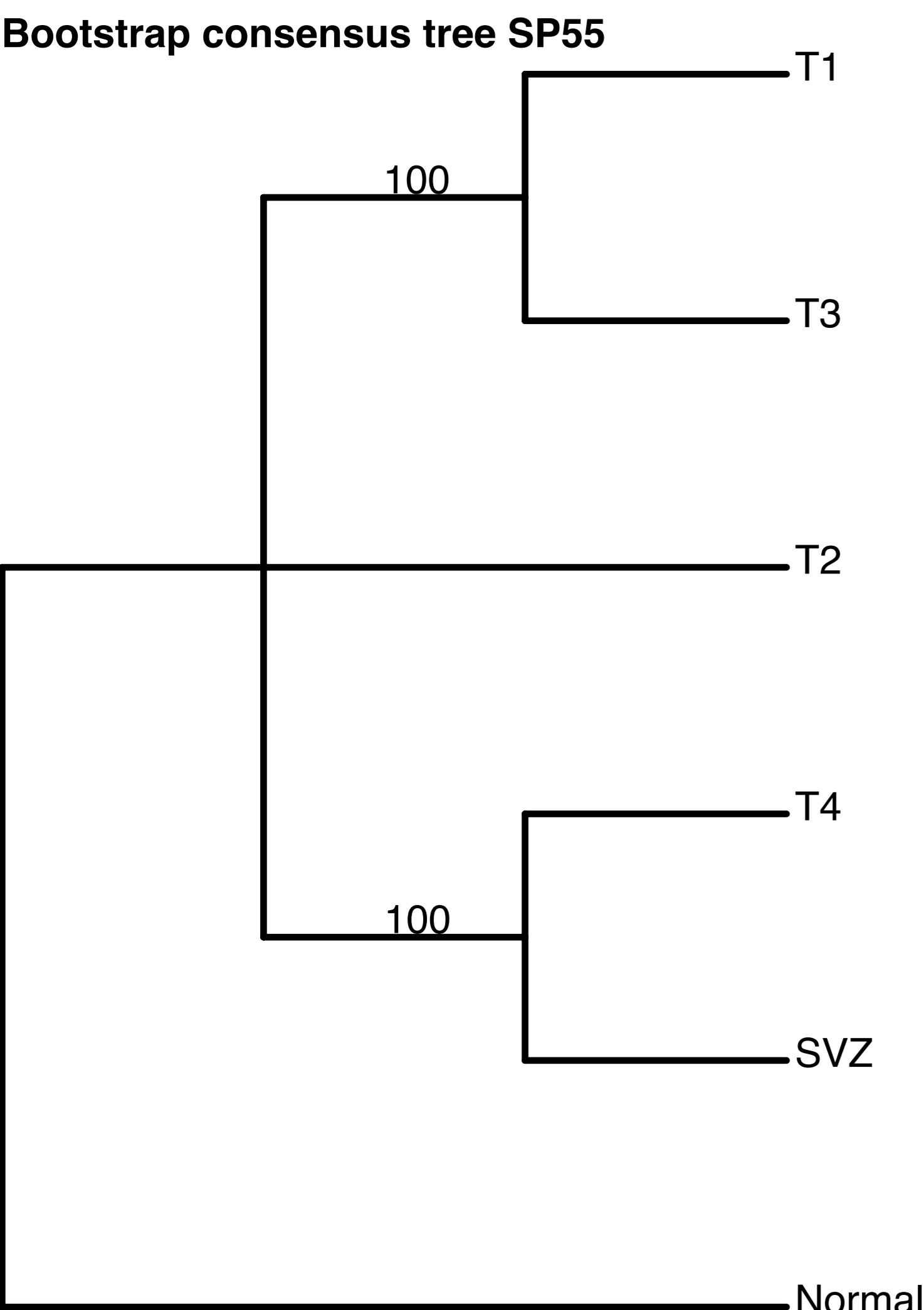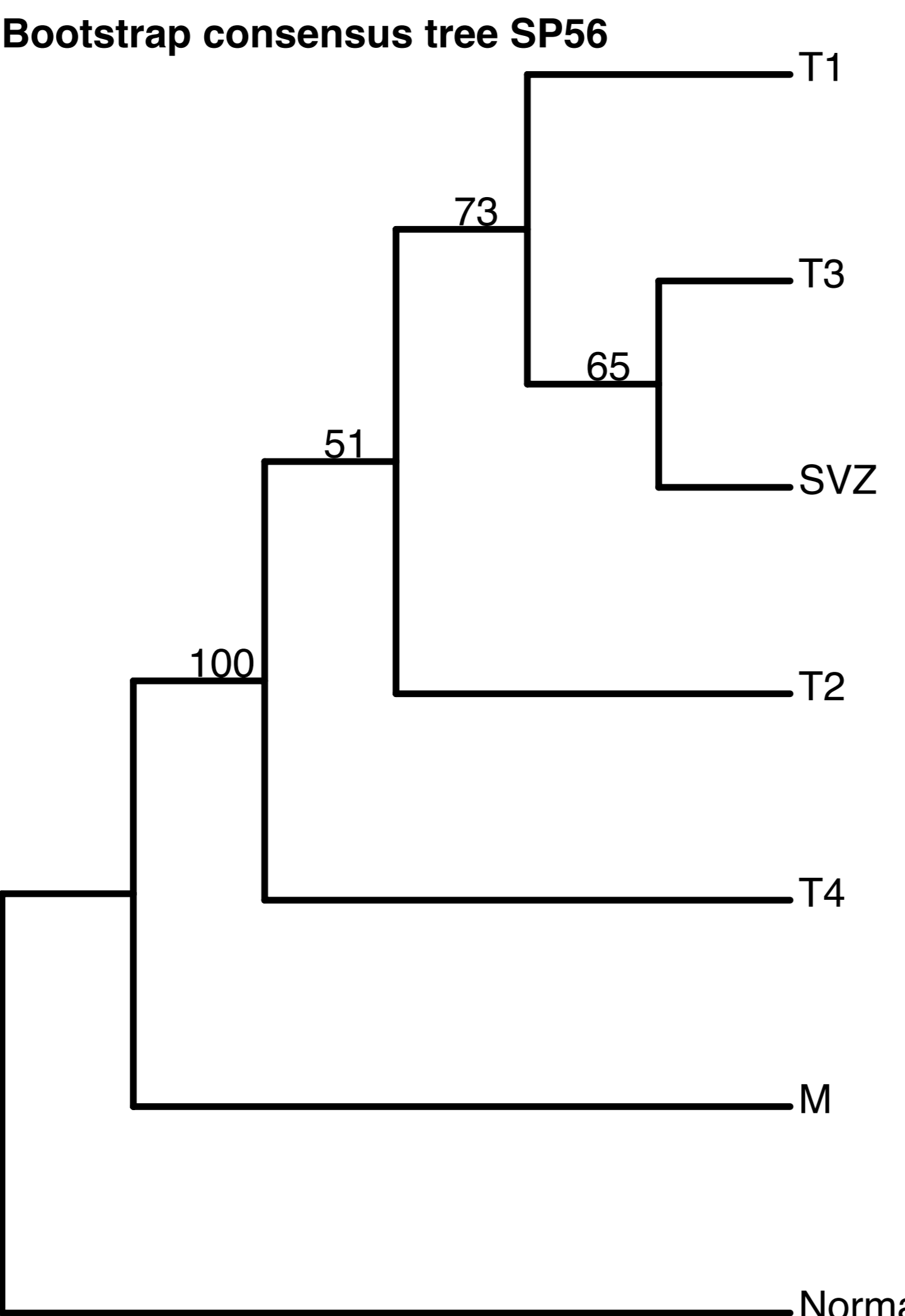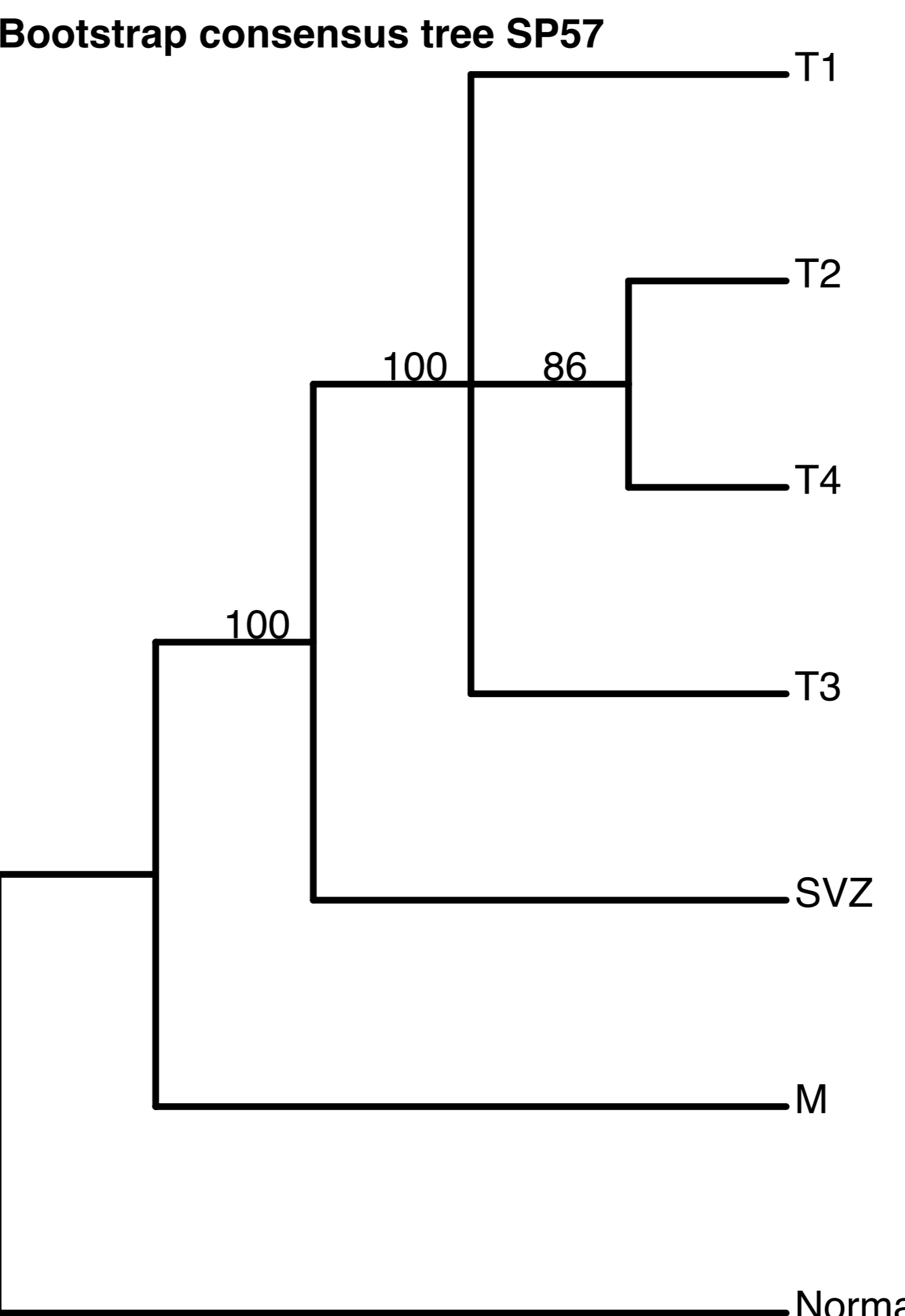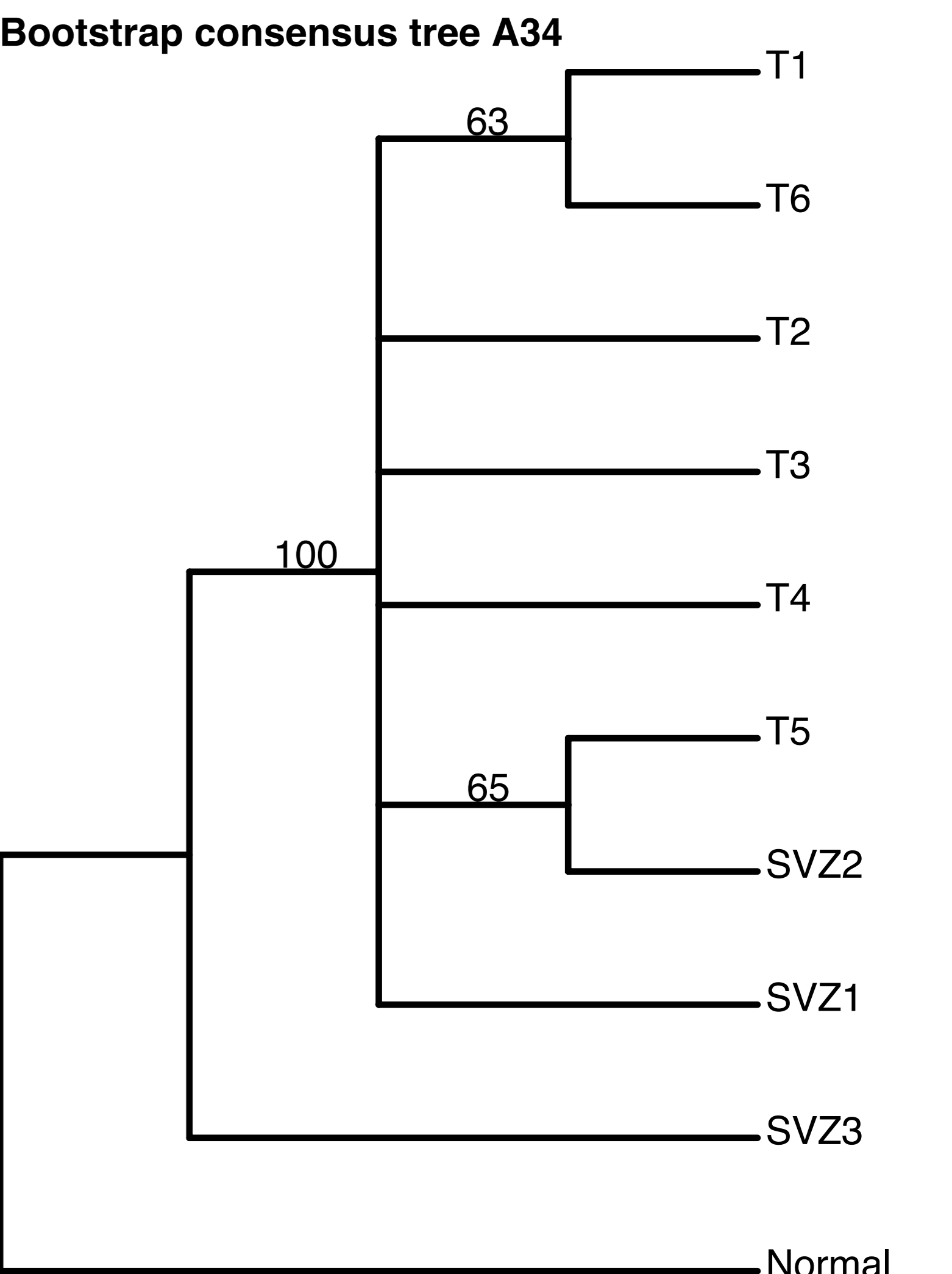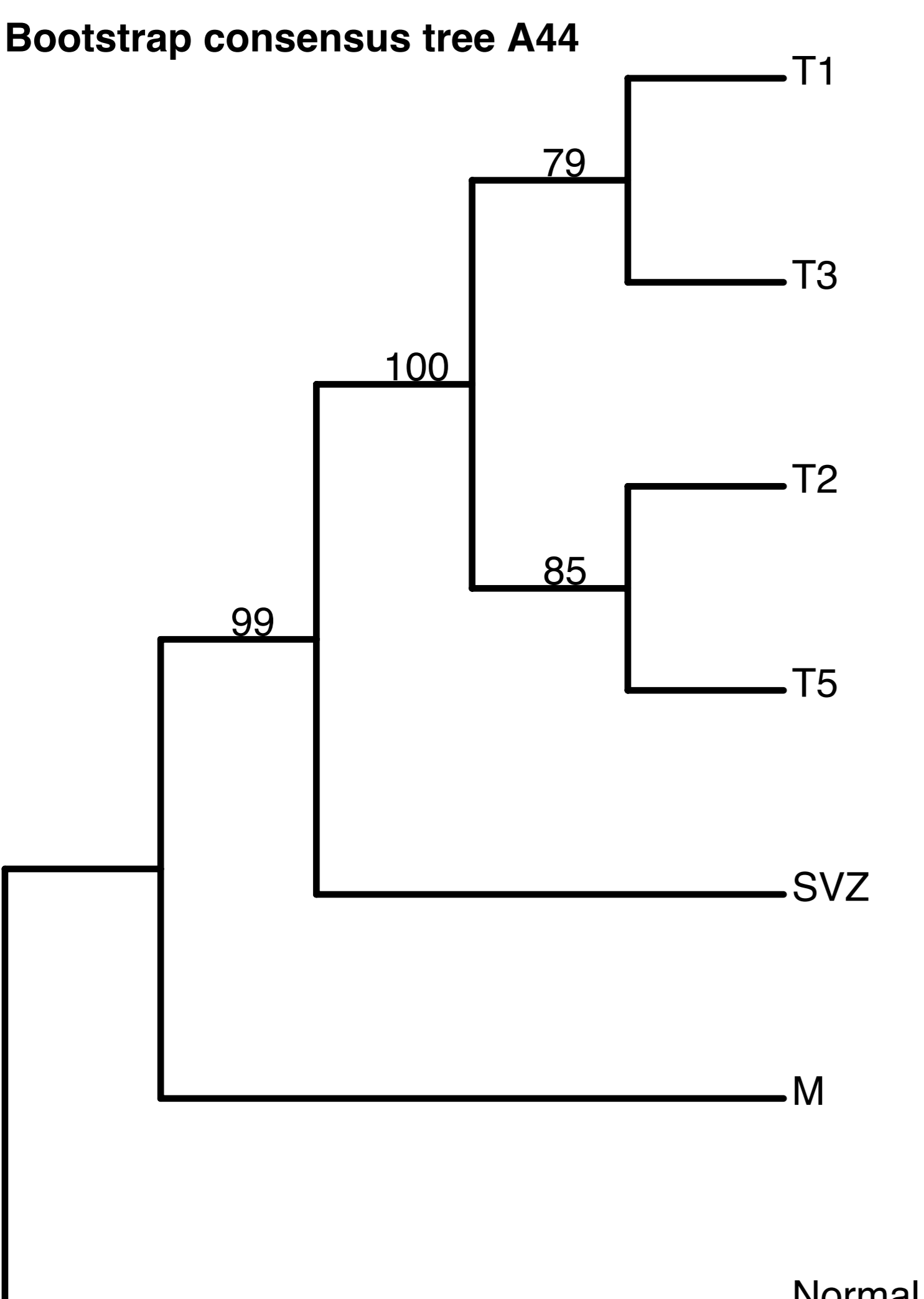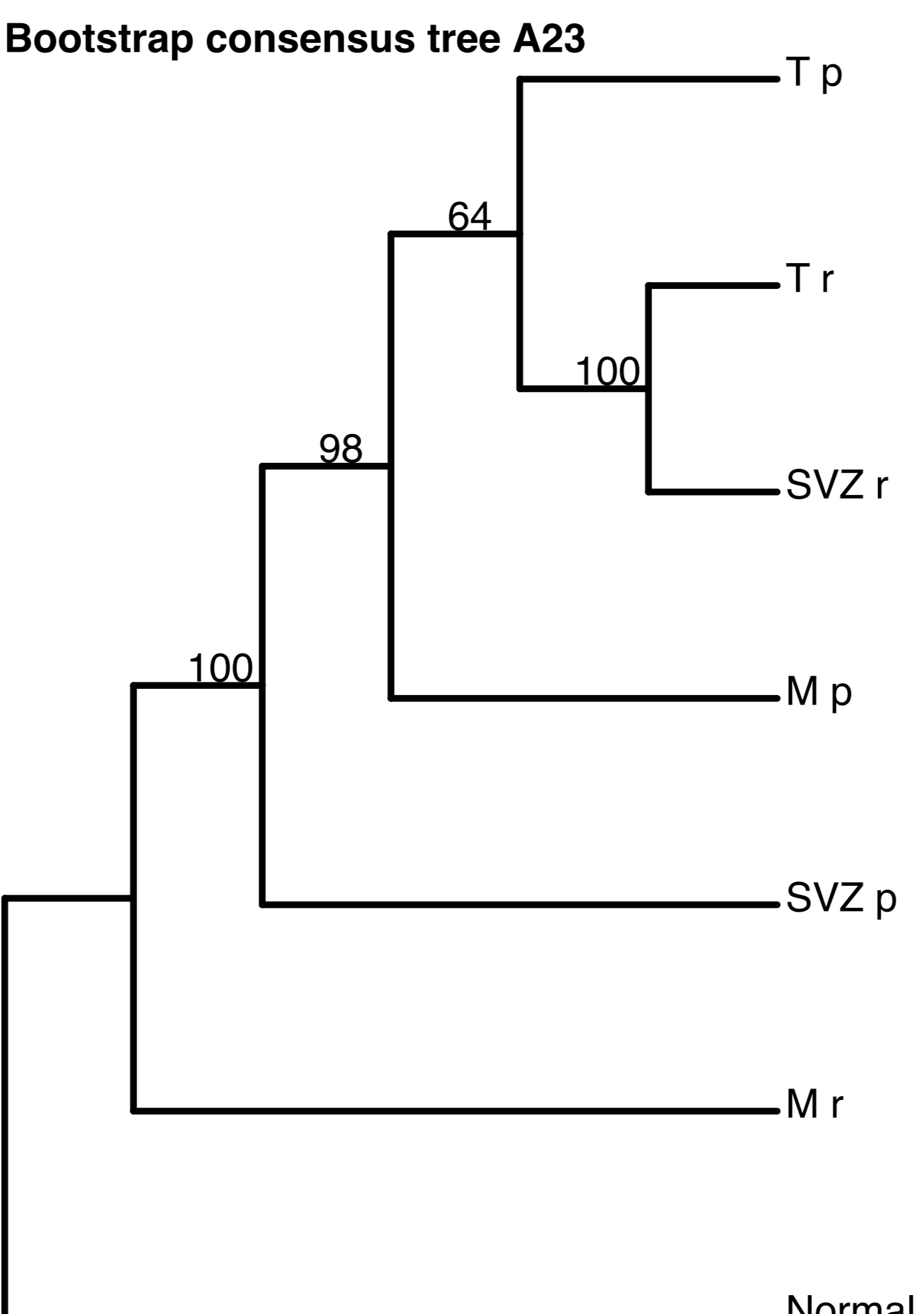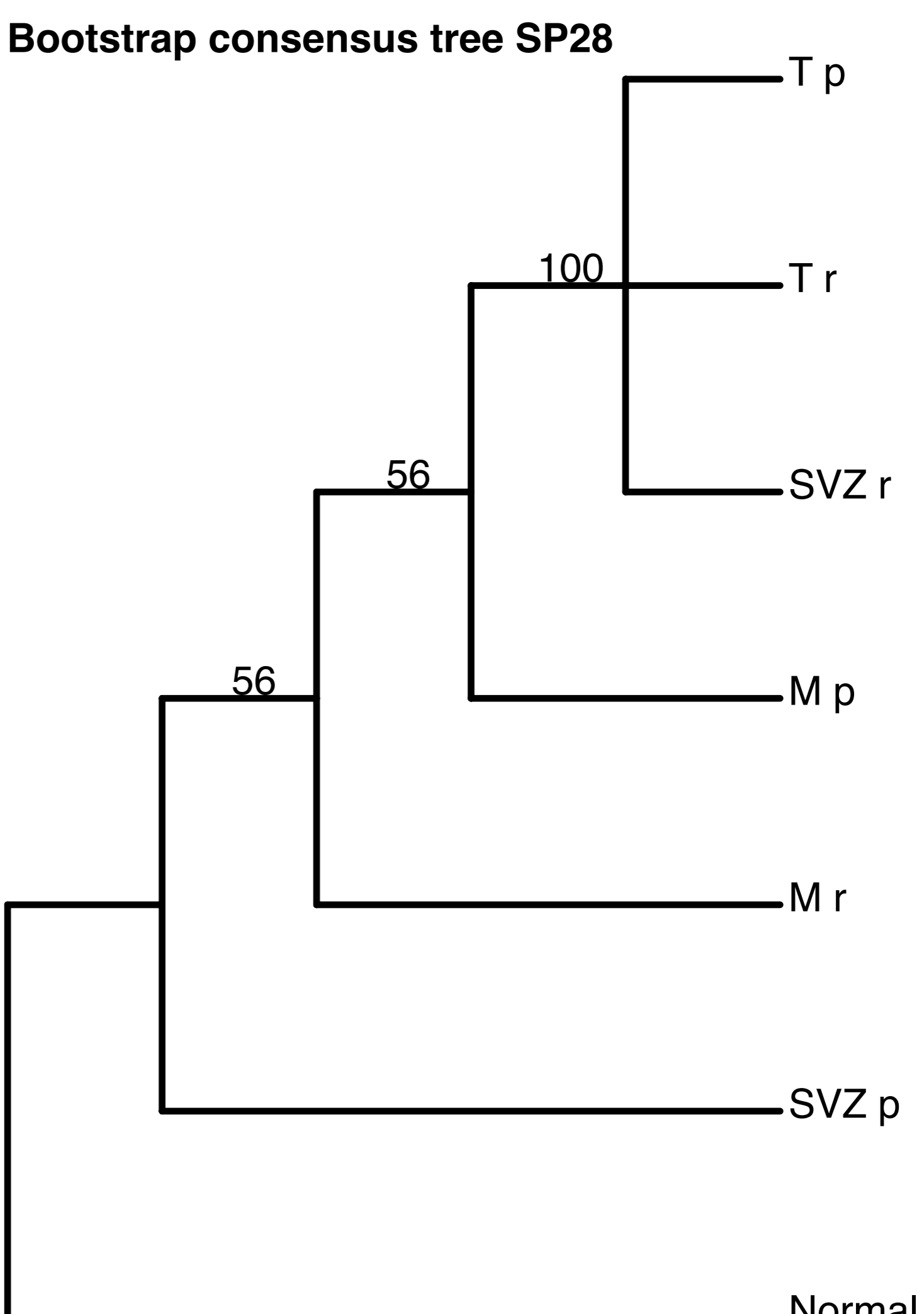

Supplement: Supplementary Data [file mdy506_supp.zip › mdy506-suppl_data/mdy506_Supplementary_Fig_S4.pdf]
