# Supplementary material for: Evolutionary dynamics of residual disease in human glioblastoma
Source: Ann Oncol. 2018 Nov 19;30(3):456–63. doi: 10.1093/annonc/mdy506 (PMC6442656; doi:10.1093/annonc/mdy506)
Supplement: Supplementary Data [file mdy506_Supplementary_Fig_S1.pdf › mdy506-suppl_data/mdy506_Supplementary_Fig_S1.pdf]

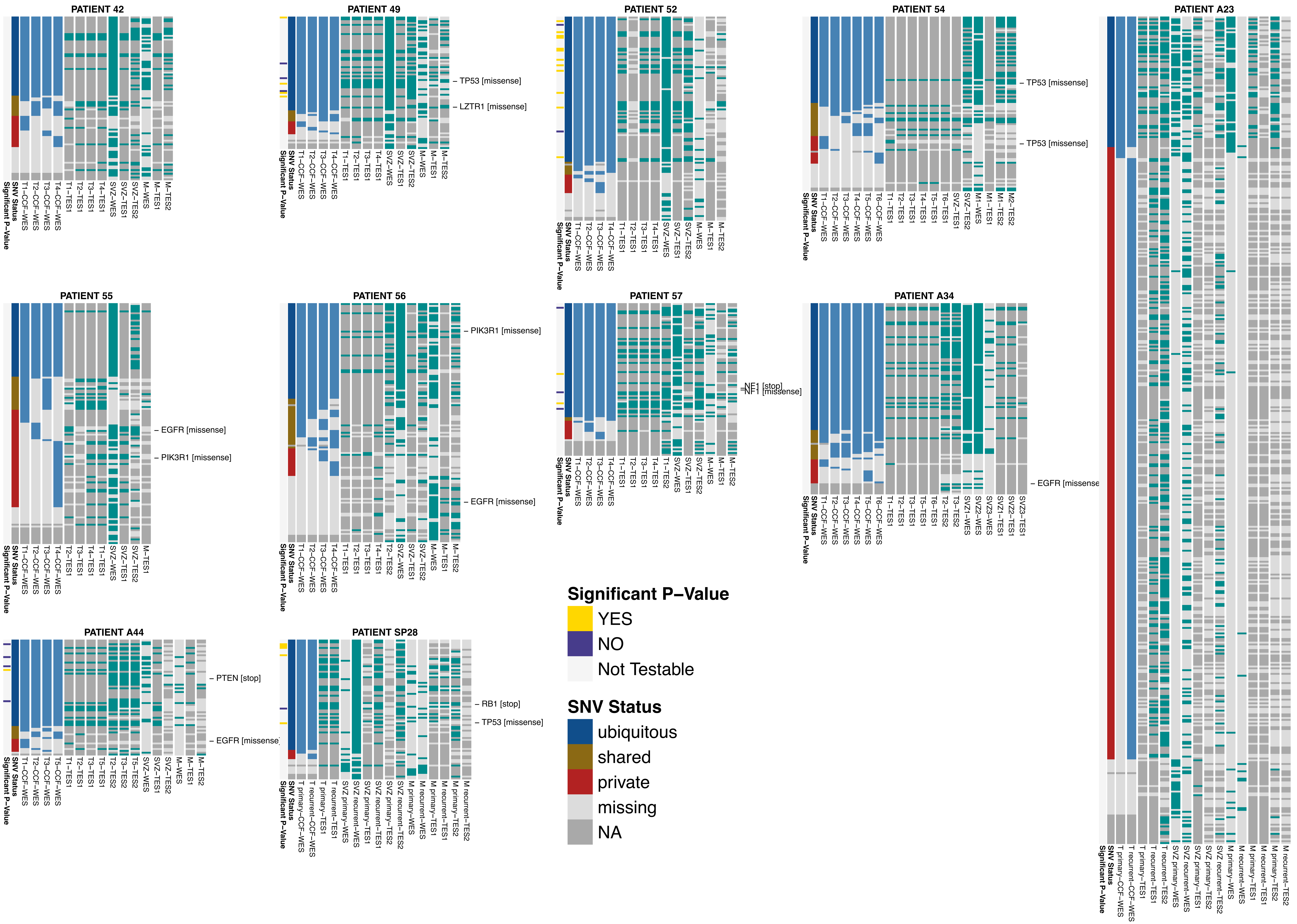

PATIENT 42
PATIENT 49
TP53 [missense]
LZTR1 [missense]
PATIENT 52
PATIENT 54
TP53 [missense]
TP53 [missense]
PATIENT A23
PATIENT 55
EGFR [missense]
PIK3R1 [missense]
PATIENT 56
PIK3R1 [missense]
EGFR [missense]
PATIENT 57
NF1 [stop]
NF1 [missense]
PATIENT A34
EGFR [missense
PATIENT A44
PTEN [stop]
EGFR [missense]
PATIENT SP28
RB1 [stop]
TP53 [missense]
Significant P–Value
YES
NO
Not Testable
SNV Status
ubiquitous
shared
private
missing
NA
